# Supplementary material for: The role of climate and islands in species diversification and reproductive-mode evolution of Old World tree frogs
Source: Commun Biol. 2022 Apr 11;5:347. doi: 10.1038/s42003-022-03292-1 (PMC9001633; doi:10.1038/s42003-022-03292-1)
Supplement: Supplementary file 1 — Supplementary Information [file 42003_2022_3292_MOESM1_ESM.pdf]

Supplementary Information for

The role of climate and islands in species diversification and reproductive-mode evolution of Old World tree frogs

Gajaba Ellepola[1,2], Marcio R. Pie[3,4], Rohan Pethiyagoda[5], James Hanken[6], Madhava Meegaskumbura[1]*

[1] College of Forestry, Guangxi Key Lab for Forest Ecology and Conservation, Guangxi University, Nanning 530004, PR China

[2] Department of Zoology, Faculty of Science, University of Peradeniya, Peradeniya, Sri Lanka

[3] Departamento de Zoologia, Universidade Federal do Paraná, Curitiba, Paraná, Brazil 81531-980

[4] Biology Department, Edge Hill University, Ormskirk, United Kingdom.

[5] Ichthyology Section, Australian Museum, Sydney, NSW 2010, Australia

[6]Museum of Comparative Zoology, Harvard University, Cambridge, MA 02138, USA

*Corresponding author

Email: madhava_m@mac.com (MM)

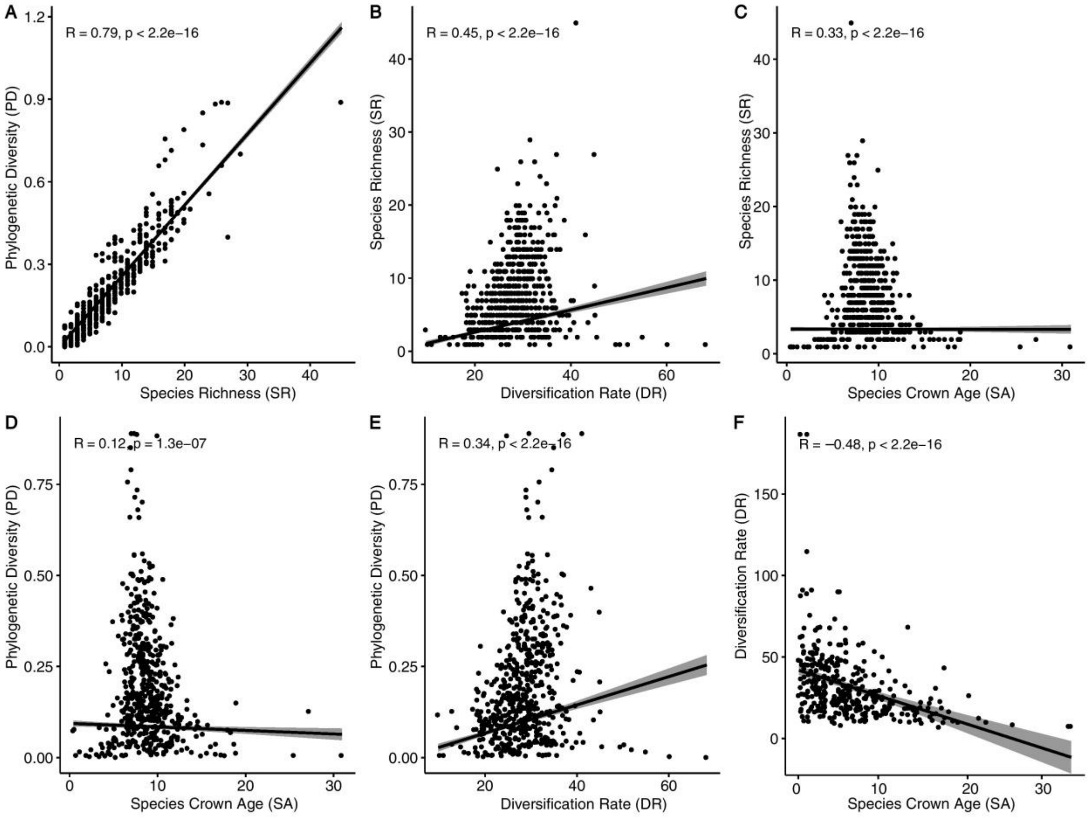

Figure 1. Correlograms depicting pairwise Spearman correlation coefficients and their statistical significance among calculated metrics SR, PD, DR and SA. All correlations are statistically significant; each metric is highly correlated with the others. This justifies the use of species richness by ranked DR quartiles (Figure 2B), which are correlated with PD and SA.

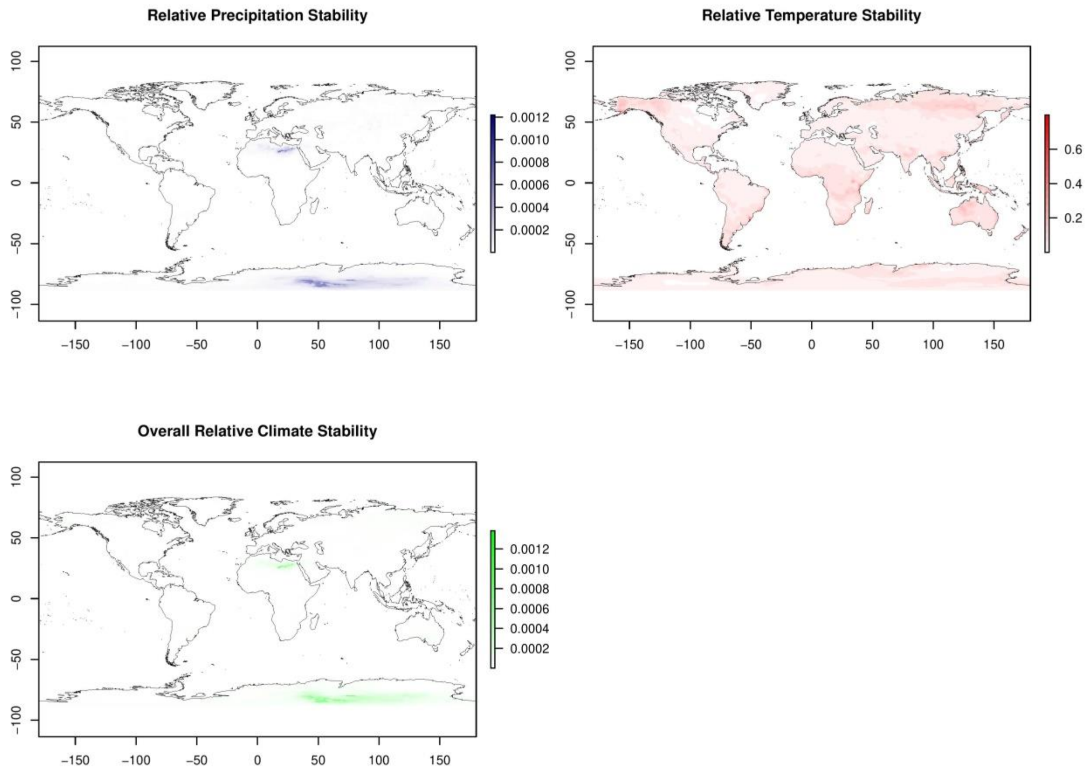

Figure 2. Relative climate stability since the Miocene (~3.3 Ma). Deeper colours denote high stability. Relative temperature since the Miocene appears stable in the regions of rhacophorid distribution.

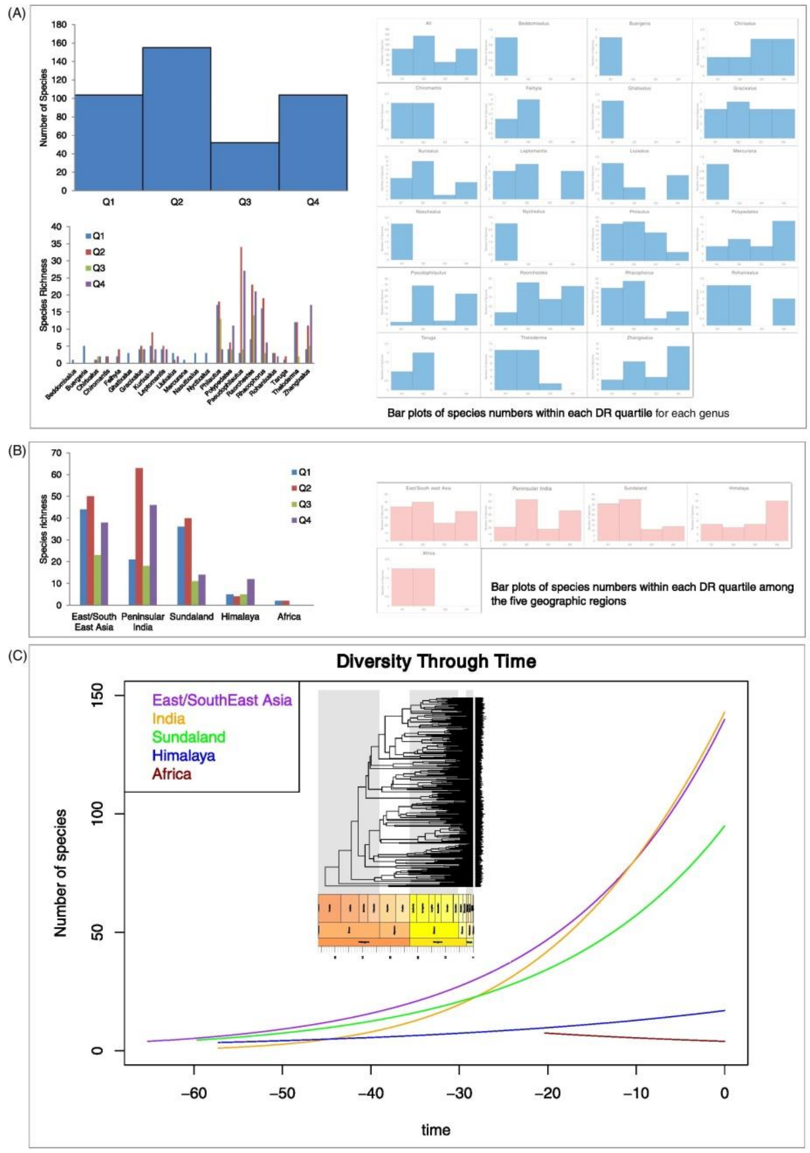

Figure 3. Temporal distribution of species accumulation of Rhacophoridae.

(A) Bar plots of species numbers within each DR quartile for each genus. The highest numbers of species accumulated during the 2nd quartile and the lowest numbers during the 3rd quartile. *Polypedates, Raorchestes, Pseudophilautus* and *Zhangixalus* each accumulated high numbers of species during the 4th quartile.
(B) Bar plots of species numbers within each DR quartile among five biogeographic regions. East/Southeast Asia and Sundaland have generated a larger number of lineages having low values of DR. The distribution of 2nd-quartile species supports a hypothesis of early dispersal and diversification events throughout East/Southeast Asia, Peninsular India and Sundaland regions. Diversification lowered during the 3rd quartile but increased again during the 4th quartile in Peninsular India, East/Southeast Asia and the Himalayas.
(C) Plot of accumulation of species through time in different geographical regions reveals spatial and temporal variation in rhacophorid diversification. Beginning gradually in all regions, diversification accelerates towards the present. The rate of species accumulation has accelerated substantially in Peninsular India since ~30 my.

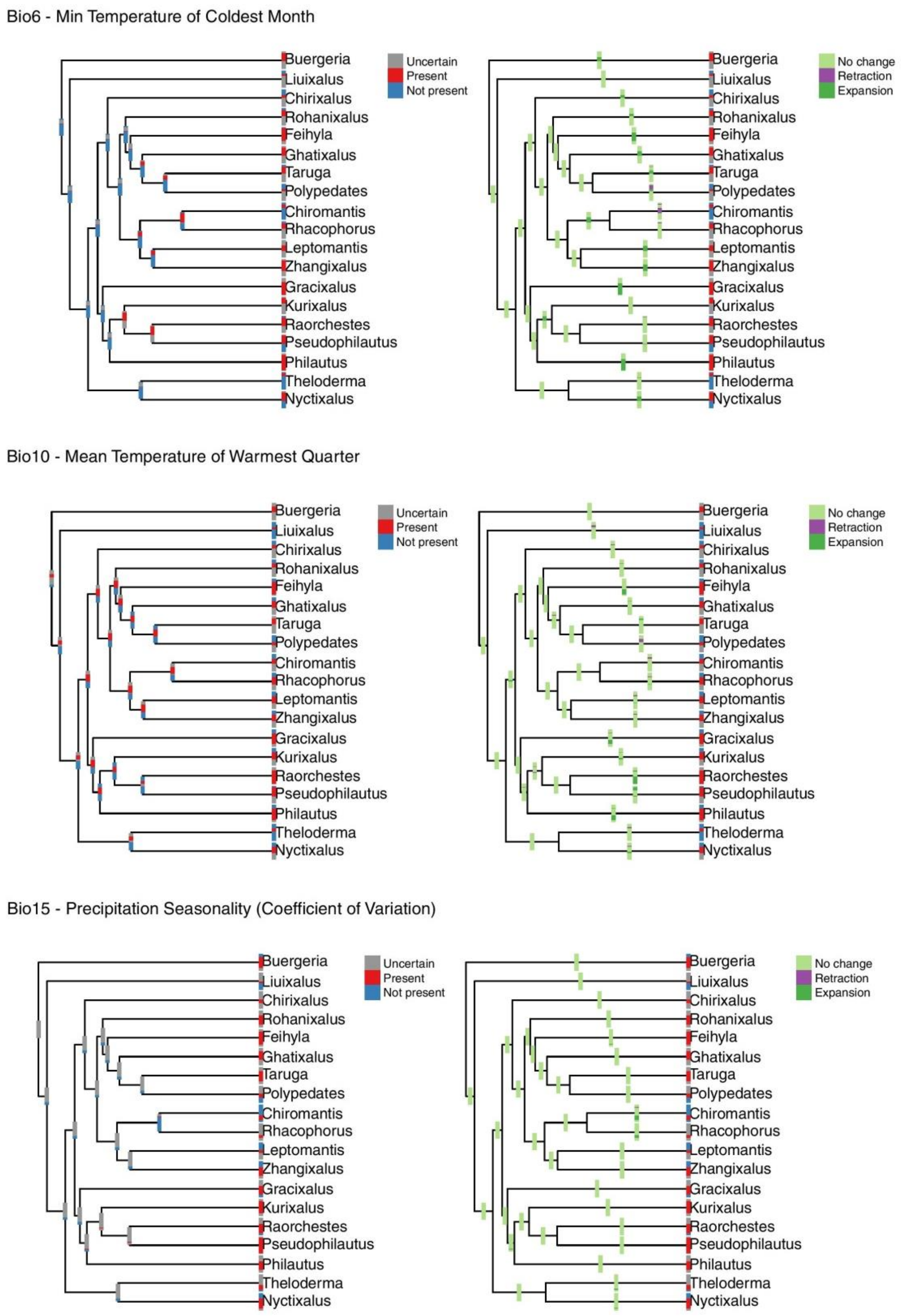

Figure 4. Results of maximum parsimony (MP) reconstructions of ecological niche evolution for major rhacophorid genera. For visualization purposes only Bio6, Bio10 and Bio15, the most dominant variables of the rhacophorid climatic niche, are shown. Left panel shows bin-based characterization of niches at tips and reconstructed bin-based values at nodes. Uncertain, present and not present denotes whether required climatic conditions are met within the accessible area (M) of the genus. Right panel shows bin-based characterization of niches at tips in comparison with reconstructed ancestors at nodes. Most of the rhacophorid genera show niche expansion, while a few show niche retraction.

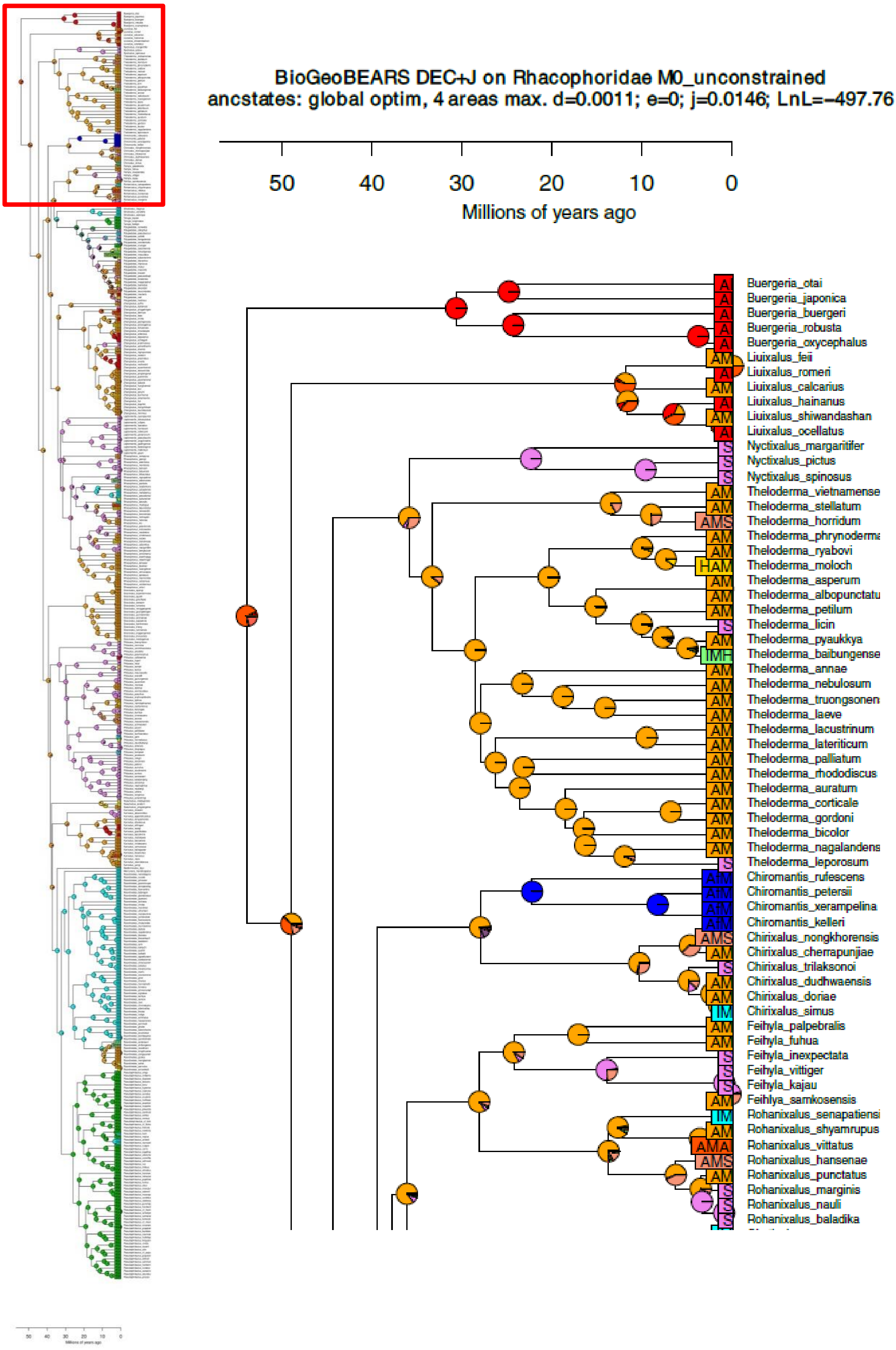

Figure 5. Mapping of mainland/island states onto the phylogeny of Rhacophoridae using the stochastic character-mapping approach.

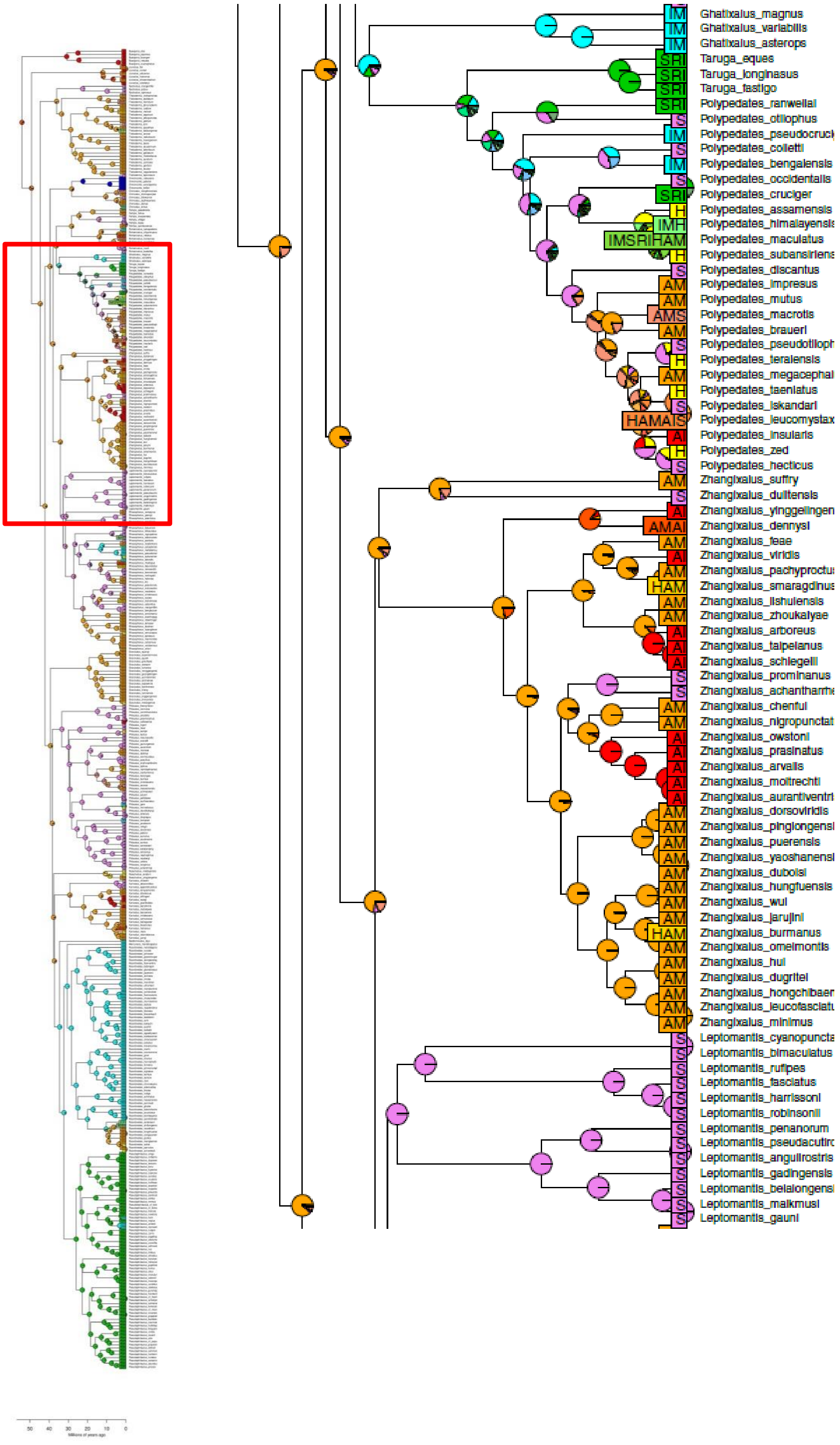

Figure 5 cont'd. Mapping of mainland/island states onto the phylogeny of Rhacophoridae using the stochastic character-mapping approach.

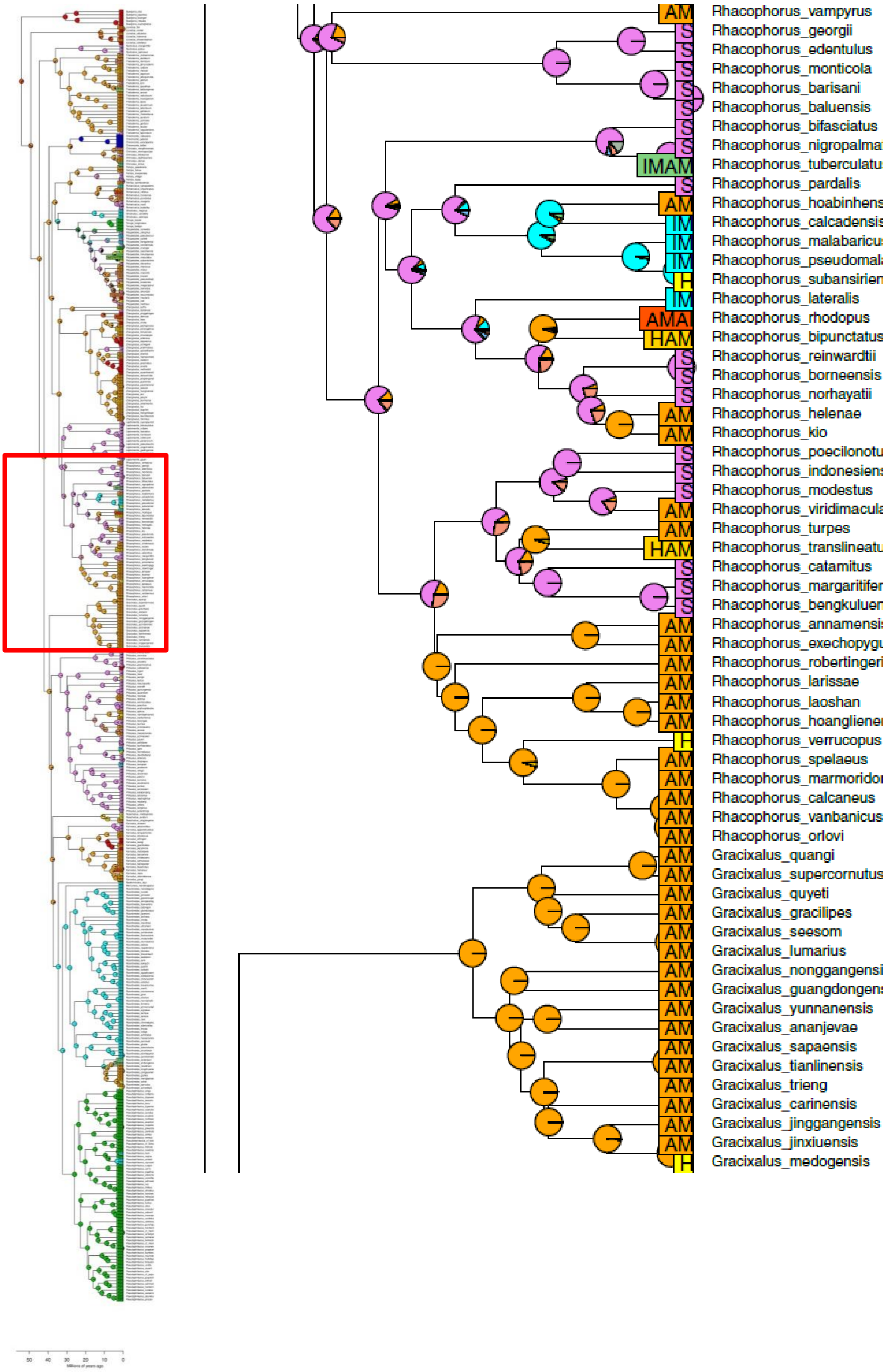

Figure 5 cont'd. Mapping of mainland/island states onto the phylogeny of Rhacophoridae using the stochastic character-mapping approach.

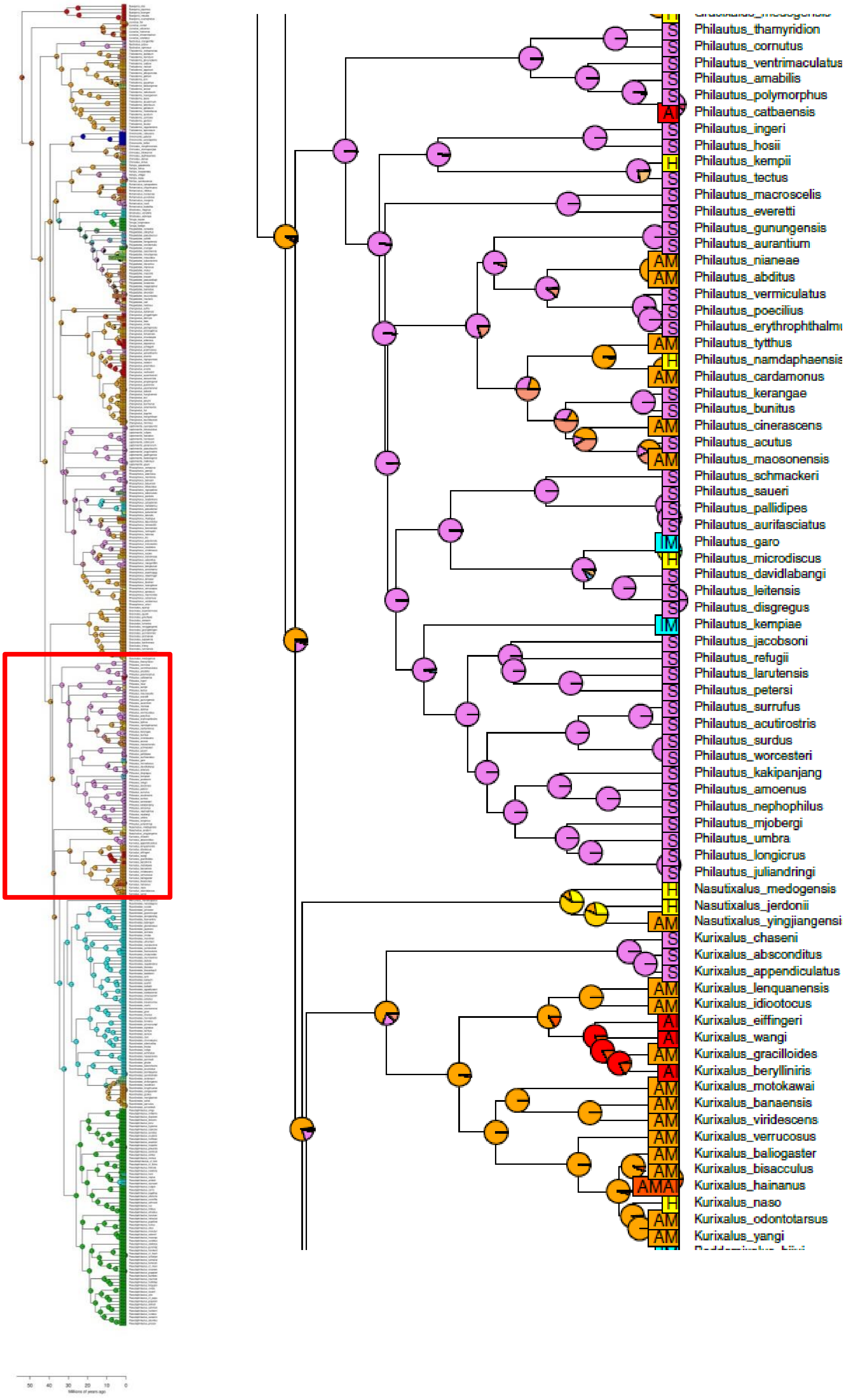

Figure 5 cont'd. Mapping of mainland/island states onto the phylogeny of Rhacophoridae using the stochastic character-mapping approach.

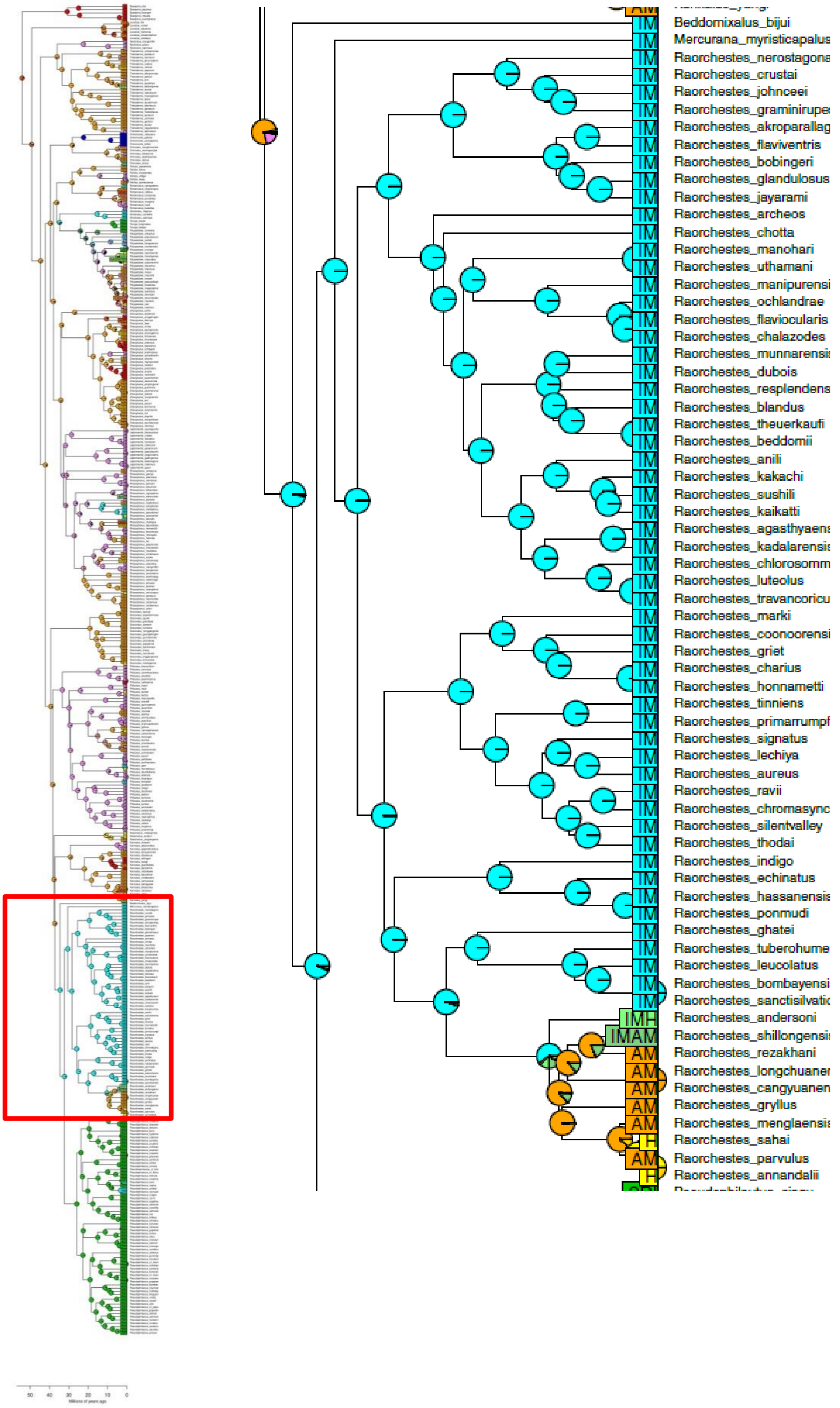

Figure 5 cont'd. Mapping of mainland/island states onto the phylogeny of Rhacophoridae using the stochastic character-mapping approach.

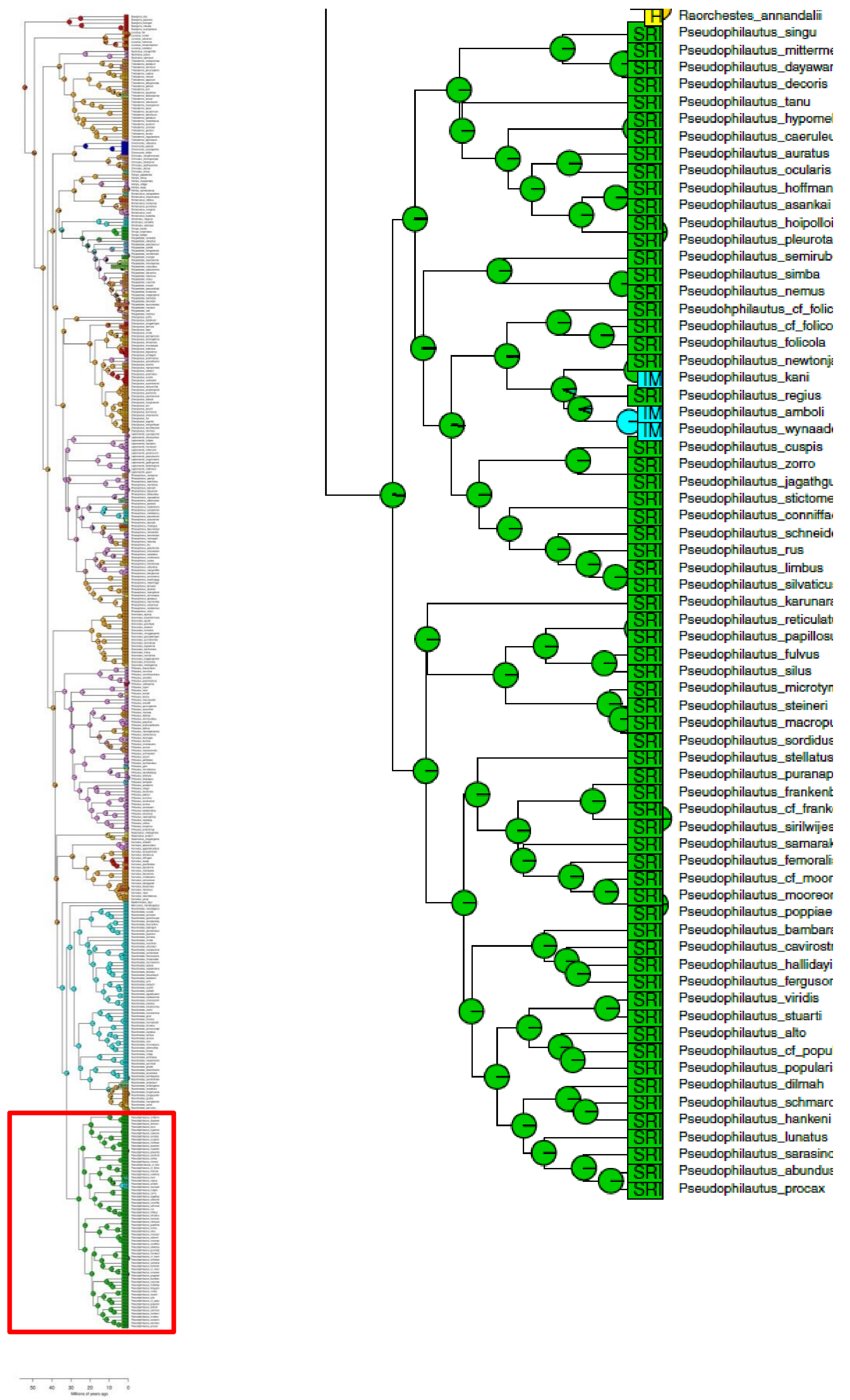

Figure 5. Mapping of mainland/island states onto the phylogeny of Rhacophoridae using the stochastic character-mapping approach. Each terminal branch represents a valid, named species. Boxes represent characters used for ancestral state reconstructions; pie charts at nodes represent posterior probabilities of character states. Species occurrences are categorized according to biogeographic areas modified from Chen et al., (2020)[18]: (1) Africa

(mainland) – AfM (dark blue); (2) Peninsular India (mainland) – IM (turquoise); (3) Sri Lanka (island) – SRI (light green); (4) East/Southeast Asia mainland – AM (orange); (5) East/Southeast Asian islands, including Japan, Taiwan, Hongkong and Hainan – AI (red); (6) Sundaland (island archipelago) – S (purple); (7) Himalayas – H (yellow). Species occurring in more than one geographic category are depicted by combinations of acronyms and mixed colors. DEC+J was recovered as the best-fitting model, indicating vicariance as well as jump dispersal events. Ancestors of many island forms have originated in mainland regions.

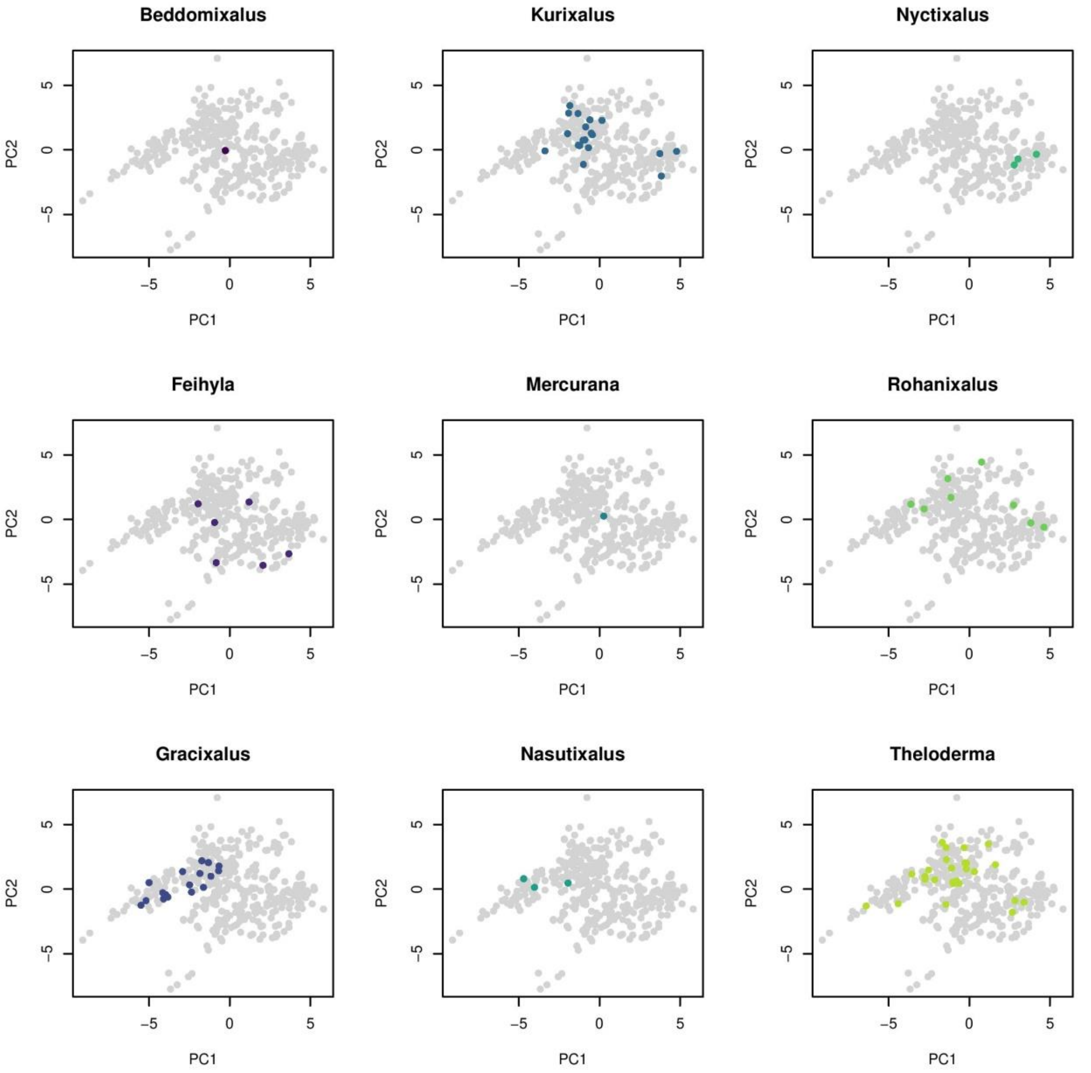

Figure 6. Climatic space defined by the first two principal component axes of climatic niches in all gel-nesting (GN) genera. The pale grey dots depict the climatic niche space occupied by all rhacophorids; the colored dots represent the occupation of the climatic niche space by different gel-nesting species. Early gel nesters are more conservative towards their ancestral climatic niche (cool-wet regions) with few species extending towards warmer climates.

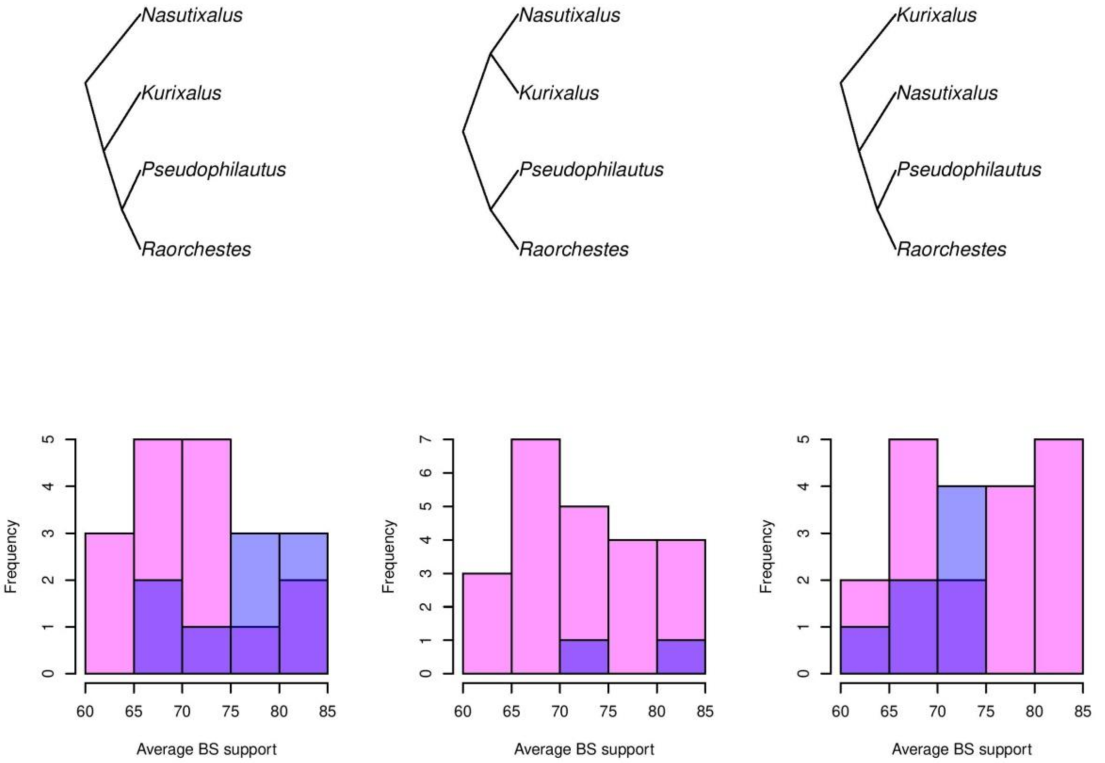

Figure 7. Three alternative phylogenetic placements of *Nasutixalus* with respect to *Kurixalus*, *Pseudophilautus* and *Raorchestes.* Histograms below each branching diagram depict the average bootstrap support (BS) of gene trees that either disagree (pink) or agree (blue) with the topology above it (purple bars indicate where the two histograms overlap). Average bootstrap support of trees that agree with the first topology (left) is significantly higher than those that disagree.

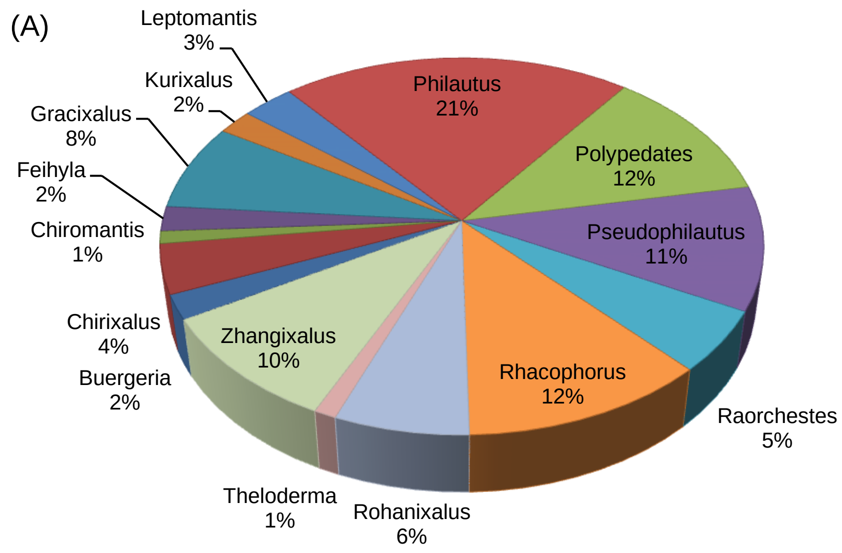

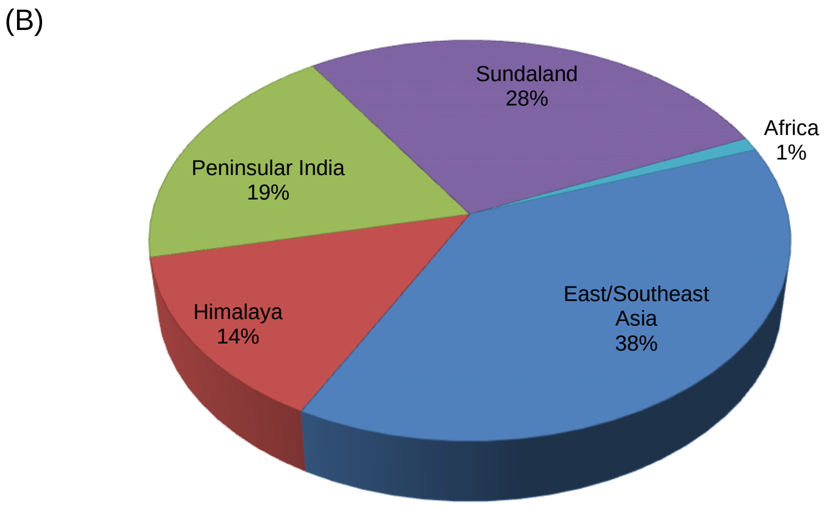

Figure 8. Summary of Rhacophoridae lacking genetic data.

(A) Percentage of species in each genus that lack genetic data (N = 94). *Philautus* accounts for the highest number of species lacking genetic data.

(B) Percentage of species in each biogeographical region that lack genetic data (N = 94). East/Southeast Asia accounts for the highest number of species lacking genetic data.

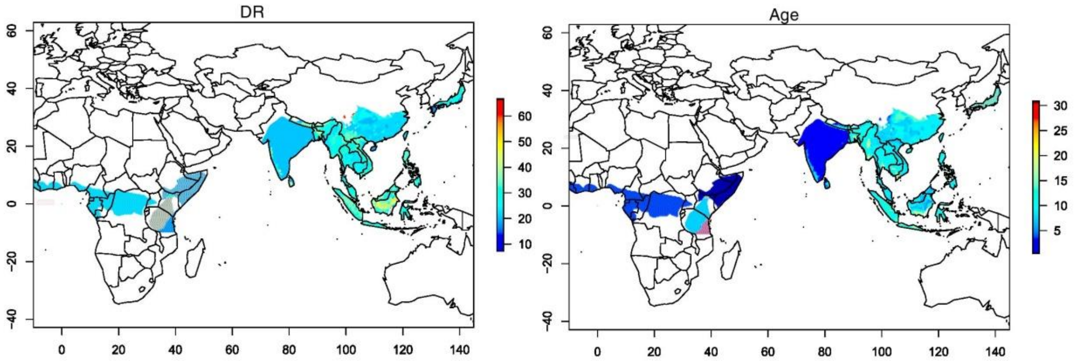

Figure 9. Spatiotemporal distribution of Rhacophoridae. (A) Map of diversification rate (DR) and species crown age in 1 °×1 ° grid cells based on presence-absence matrices derived from species geographic distributions. These metrics vary spatially and temporally during rhacophorid diversification such that high DR are observed in Borneo islands, Peninsular Malaysia, Vietnam, Yunnan-Guizhou plateau area, Western Ghats of India and Sri Lanka. These geographic regions also contain the oldest and youngest rhacophorid lineages. Highest species richness and phylogenetic diversity are also recorded in these (see Fig. 2a).

Table 1. Published studies of rhacophorid phylogeny based on more than 12 species each.

Reference	Number of species	Source
**Abraham et al., 2013**	62	Abraham, R.K., Pyron, R.A., Ansil, B.R., Zachariah, A.A., Zachariah, A.A. (2013). Two novel genera and one new species of treefrog (Anura: Rhacophoridae) highlight cryptic diversity in the Western Ghats of India. *Zootaxa* 3640, 177–199. https://doi.org/10.11646/zootaxa.3640.2.3.
**Biju and Bossuyt, 2008**	34	Biju, S.D. and Bossuyt, F. (2009). Systematics and phylogeny of *Philautus* Gistel, 1848 (Anura, Rhacophoridae) in the Western Ghats of India, with descriptions of 12 new species. *Zoological Journal of the Linnean Society* 155: 374-444. doi:10.1111/j.1096-3642.2008.00466.x
**Biju et al., 2020**	91	Biju, S. D. *et al.* (2020). New insights on the systematics and reproductive behaviour in tree frogs of the genus *Feihyla*, with description of a new related genus from Asia (Anura, Rhacophoridae). *Zootaxa* **4878**, 1–55. DOI: 10.11646/zootaxa.4878.1.1
**Boruah et al., 2018**	16	Boruah, B., Raj, P., Dutta, S., & Das, A. (2018). Redescription and geographic distribution of *Raorchestes shillongensis* (Anura: Rhacophoridae) from Meghalaya, Northeast India. Phyllomedusa: *Journal of Herpetology* 17(1): 3-20. https://doi.org/10.11606/issn.2316-9079.v17i1p3-20
**Chan et al., 2018**	247	Chan, K.O, Grismer, L.L, Brown, R.M. (2018). Comprehensive multi-locus phylogeny of Old World tree frogs (Anura: Rhacophoridae) reveals taxonomic uncertainties and potential cases of over- and underestimation of species diversity. *Molecular Phylogenetics and Evolution* 127:1010-1019. doi:10.1016/j.ympev.2018.07.005
**Chen et al., 2020**	66	Chen, J. M., Prendini, E., Wu, Y. H., Zhang, B. L., Suwannapoom, C., Chen, H.M., Jin, J.Q., Lemmon, E.M., Lemmon, A.R., Stuart, B.L., Raxworthy, C.J., Murphy, R.W., Yuan, Z.Y. and Che. J. (2020). An integrative phylogenomic approach illuminates the evolutionary history of Old World tree frogs (Anura: Rhacophoridae). *Molecular Phylogenetics and Evolution* 145 106724. https://doi.org/10.1016/j.ympev.2019.106724
**Dang et al., 2015**	37	Dang, N. X., Sun, F. H., Lv, Y. Y., Zhao, B. H., Wang, J. C., Murphy, R. W., Wang, W. Z & Li, J. T. (2016) DNA barcoding and the identification of tree frogs (Amphibia: Anura: Rhacophoridae), *Mitochondrial DNA Part A*, 27:4, 2574-2584, DOI: 10.3109/19401736.2015.1041113
**Dehling et al., 2016**	25	Dehling, J.M., Matsui, M. and Imbun, P.Y. (2016). A new small montane species of *Philautus* (Amphibia: Anura: Rhacophoridae) from Gunung Kinabalu, Sabah, Malaysia (Borneo). *Salamandra* 52(2): 77–90
**Dever 2017**	26	Dever, A. J. (2017). A new cryptic species of the *Theloderma asperum* complex (Anura: Rhacophoridae) from Myanmar. *Journal of Herpetology* 51(3): 425-436. https://doi.org/10.1670/17-026
**Grosjean et al., 2008**	48	Grosjean, S., Delorme, M., Dubois, A. and Ohler, A. (2008), Evolution of reproduction in the Rhacophoridae (Amphibia, Anura). *Journal of Zoological Systematics and Evolutionary Research* 46: 169-176. doi:10.1111/j.1439-0469.2007.00451.x

**Hertwig et al., 2013**	91	Hertwig, S.T., Schweizer, M., Das, I., Haas, A. (2013). Diversification in a biodiversity hotspot - the evolution of Southeast Asian rhacophorid tree frogs on Borneo (Amphibia: Anura: Rhacophoridae). *Molecular Phylogenetics and Evolution* 68: 567–581. https://doi.org/10.1016/j.ympev.2013.04.001.
**Jiang et al., 2019**	58	Jiang, D, Jiang, Ke., Ren, J., Wu, J. and Li, J. (2019). Resurrection of the genus *Leptomantis*, with description of a new genus to the family Rhacophoridae (Amphibia: Anura). *Asian Herpetological Research* 10(1): 1-12. [doi:10.16373/j.cnki.ahr.180058]
**Li et al., 2008**	46	Li, J., Che, J., Bain, R. H., Zhao, E. and Zhang, Y (2008). Molecular phylogeny of Rhacophoridae (Anura): a framework of taxonomic reassignment of species within the genera *Aquixalus*, *Chiromantis*, *Rhacophorus* and *Philautus*. *Molecular Phylogenetics and Evolution* 48: 302–312. https://doi.org/10.1016/j.ympev.2008.03.023.
**Li et al., 2009**	~50	Li, J.T., Che, J., Murphy, R.W., Zhao, H., Zhao, E.M., Rao, D.Q., Zhang, Y.P., 2009. New insights to the molecular phylogenetics and generic assessment in the Rhacophoridae (Amphibia: Anura) based on five nuclear and three mitochondrial genes, with comments on the evolution of reproduction. *Molecular Phylogenetics and Evolution* 53: 509–522. https://doi.org/10.1016/j.ympev.2009.06.023.
**Li et al., 2012**	52	Li, J.-T., Li, Y., Murphy, R.W., Rao, D.-Q. and Zhang, Y.-P. (2012). Phylogenetic resolution and systematics of the Asian tree frogs, *Rhacophorus* (Rhacophoridae, Amphibia). *Zoologica Scripta* 41: 557-570. doi:10.1111/j.1463-6409.2012.00557.x
**Li et al., 2013**	114	Li, J.-T., Y. Li, S. Klaus, D.-Q. Rao, D. M. Hillis, and Y.-P. Zhang. (2013). Diversification of rhacophorid frogs provides evidence for accelerated faunal exchange between India and Eurasia during the Oligocene. *Proceedings of the National Academy of Sciences of the United States of America* 110: 344 – 3446.https://doi.org/10.1073/pnas.1300881110.
**Lv et al., 2018**	12	Lv, Y-Y., He, K., Klaus, S., Brown, R.M., Li, J-T. (2018). A comprehensive phylogeny of the genus *Kurixalus* (Rhacophoridae, Anura) sheds light on the geographical range evolution of frilled swamp treefrogs. *Molecular Phylogenetics and Evolution* 121:224-232. doi: https://doi.org/10.1016/j.ympev.2017.09.019
**Matsui et al., 2015**	15	Matsui, M., Khonsue, W., Panha, S. and Eto, K. (2015). A new tree frog of the genus *Gracixalus* from Thailand (Amphibia: Rhacophoridae). *Zoological Science* 32(2): 204-210. http://dx.doi.org/10.2108/zs140238
**Meegaskumbura et al., 2002**	>19	Meegaskumbura, M., Bossuyt, F., Pethiyagoda, R., Manamendra-Arachchi, K., Bahir, M., Milinkovitch, M. C and Schneider, C. J. (2002). Sri Lanka: An amphibian hot spot. *Science* 298 (5592): 379. DOI: 10.1126/science.298.5592.379
**Meegaskumbura et al., 2010**	28	Meegaskumbura, M., Meegaskumbura, S., Bowatte, G., Manamendra-Arachchi, K., Pethiyagoda, R., Hanken, J. and Schneider, C.J., 2011. *Taruga* (Anura: Rhacophoridae), a new genus of foam-nesting tree frogs endemic to Sri Lanka. *Ceylon Journal of Science (Biological Sciences)* 39(2): 75–94. DOI: http://doi.org/10.4038/cjsbs.v39i2.2995
**Meegaskumbura et al., 2015**	~70	Meegaskumbura, M., Senevirathne, G., Biju, S.D., Garg, S., Meegaskumbura, S., Pethiyagoda, R., Hanken, J. and Schneider, C.J. (2015). Patterns of

		reproductive-mode evolution in Old World tree frogs (Anura, Rhacophoridae). *Zoologica Scripta* 44(5): 1–14. https://doi.org/10.1111/zsc.12121.
**Meegaskumbura et al., 2019**	67	Meegaskumbura, M., Senevirathne, G., Manamendra-Arachchi, K., Pethiyagoda, R., Hanken, J. and Schneider, C.J. (2019). Diversification of shrub frogs (Rhacophoridae Pseudophilautus) in Sri Lanka-Timing and geographic context. *Molecular Phylogenetics and Evolution* 132: 14–24. https://doi.org/10.1016/j.ympev.2018.11.004
**Nguyem et al., 2020**	38	Nguyen, T. V., Duong, T. V., Luu, K. T and Poyarkov, N. A. (2020). A new species of *Kurixalus* (Anura: Rhacophoridae) from northern Vietnam with comments on the biogeography of the genus. *Journal of Natural History* 54: 1-4, 195-223. DOI: 10.1080/00222933.2020.1728411
**Padhye et al., 2013**	34	Padhye, A., Sayyed, A., Jadhav, A., and Dahanukar, N. (2013). *Raorchestes ghatei*, a new species of shrub frog (Anura: Rhacophoridae) from the Western Ghats of Maharashtra, India. *Journal of Threatened Taxa* 5(15): 4913-4931. https://doi.org/10.11609/JoTT.o3702.4913-31
**Pan et al., 2017**	58	Pan, T., Zhang, Y., Wang, H., Wu, J., Kang, X., Qian, L., Chen, J., Rao, D., Jiang, J. and Zhang, B. (2017). The reanalysis of biogeography of the Asian tree frog, *Rhacophorus* (Anura: Rhacophoridae): geographic shifts and climatic change influenced the dispersal process and diversification. *PeerJ* 5: e3995. https://doi.org/10.7717/peerj.3995.
**Poyarkov et al., 2018**	~30	Poyarkov, N. A., Jr., Kropachev, I. I., Gogoleva, S. S and Orlov, N. L (2018). A new species of the genus *Theloderma* Tschudi, 1838 (Amphibia: Anura: Rhacophoridae) from Tay Nguyen Plateau, central Vietnam. *Zoological Research* 39(3): 158-184. doi: 10.24272/j.issn.2095-8137.2018.018
**Pyron and Wiens, 2011**	108	Pyron, A.R. and Wiens, J.J. (2011). A large-scale phylogeny of Amphibia including over 2800 species, and a revised classification of extant frogs, salamanders, and caecilians. *Molecular Phylogenetics and Evolution* 61: 543–583. https://doi.org/10.1016/j.ympev.2011.06.012.
**Rowley et al., 2020**	20	Rowley, J.J.L., Le, D.T.T., Hoang, H.D., Cao, T.T. and Dau, V.Q (2020). A new species of phytotelm breeding frog (Anura: Rhacophoridae) from the Central Highlands of Vietnam. *Zootaxa* 4779 (3): 341–354. https://doi.org/10.11646/zootaxa.4779.3.3
**Vijayakumar et al., 2016**	~60	Vijayakumar, S.P, Menezes, R.C, Jayarajan, A. and Shanker, K. (2016). Glaciations, gradients, and geography: multiple drivers of diversification of bush frogs in the Western Ghats Escarpment. *Proceedings of the Royal Society B* 283:20161011.http://dx.doi.org/10.1098/rspb.2016.1011
**Vijayakumar et al., 2018**	57	Vijayakuma, S., Dinesh, K., Prabhu, M., and Shanker, K. (2014). Lineage delimitation and description of nine new species of bush frogs (Anura: *Raorchestes*, Rhacophoridae) from the Western Ghats Escarpment. *Zootaxa* 3893: 4. http://dx.doi.org/10.11646/zootaxa.3893.4.1
**Wilkinson et al., 2002**	28	Wilkinson, J. A., Drewes, R. C and Tatum, O. L. (2002). A molecular phylogenetic analysis of the family Rhacophoridae with an emphasis on the Asian and African genera. *Molecular Phylogenetics and Evolution* 24: 265–

		273.doi:10.1016/s1055-7903(02)00212-9
**Wostle et al., 2017**	50	Wostl E., Riyanto, A., Hamidy, A., Kurniawan, N, Smith, E.N. and Harvey, M.B. (2017). A taxonomic revision of the *Philautus* (Anura: Rhacophoridae) of Sumatra with the description of four new species, *Herpetological Monographs* 31(1): 98-141. https://doi.org/10.1655/HERPMONOGRAPHS-D-16-00007
**Yu et al., 2008**	23	Yu, G., Rao, D., Yang, J. and Zhang, M. (2008). Phylogenetic relationships among Rhacophorinae (Rhacophoridae, Anura, Amphibia), with an emphasis on the Chinese species. *Zoological Journal of the Linnean Society* 153: 733–749.
**Yu et al., 2009**	57	Yu, G. H., Rao, D. Q., Zhang, M. W. & Yang, J. X. (2009). Reexamination of the phylogeny of Rhacophoridae (Anura) based on mitochondrial and nuclear DNA. *Molecular Phylogenetics and Evolution* 50: 571–579.
**Yu et al., 2020**	24	Yu, G. H., Du, L. N. Wang, J. S. Rao, D. Q., Wu, Z. J and Yang J. X. (2020). From mainland to islands: colonization history in the tree frog *Kurixalus* (Anura: Rhacophoridae). *Current Zoology*, 66(6): 667–675. https://doi.org/10.1093/cz/zoaa023
**Chan et al., 2020**	35	Chan, K.O., Hutter, C.R., Wood, P.L., Grismer, L.L and Brown, R.M. (2020). Target-capture phylogenomics provide insights on gene and species tree discordances in Old World treefrogs (Anura: Rhacophoridae). *Proceedings of the Royal Society B*, 287: 20202102. doi:10.1098/rspb.2020.2102

Table 2. Loadings on the first three principal component axes of bioclimatic data associated with the occurrence of rhacophorid species. PC1, PC2 and PC3 explain 40.5%, 23.9% and 15.5% of the variance, respectively. PC1 is associated with variation in temperature (BIO 6, BIO9, BIO11), PC2 with rainfall and temperature (BIO 5, BIO 8, BIO 10, BIO15), and PC3 with high summer temperature (BIO 5, BIO10, BIO 11).

**Variable**	**PC1**	**PC2**	**PC3**
BIO1 = Annual Mean Temperature	0.29	0.25	0.14
BIO2 = Mean Diurnal Range (Mean of monthly (max temp - min temp))	-0.13	0.06	0.1
BIO3 = Isothermality (BIO2/BIO7) (×100)	0.27	-0.23	-0.02
BIO4 = Temperature Seasonality (standard deviation ×100)	-0.28	0.17	0.06
BIO5 = Maximum Temperature of the Warmest Month	0.16	0.36	0.21
BIO6 = Minimum Temperature of the Coldest Month	0.35	0.06	0.06
BIO7 = Temperature Annual Range (BIO5-BIO6)	-0.3	0.19	0.08
BIO8 = Mean Temperature of the Wettest Quarter	0.15	0.35	0.2
BIO9 = Mean Temperature of the Driest Quarter	0.34	0.11	0.07
BIO10 = Mean Temperature of the Warmest Quarter	0.17	0.36	0.19
BIO11 = Mean Temperature of the Coldest Quarter	0.34	0.1	0.08
BIO12 = Annual Precipitation	0.18	0.06	-0.47
BIO13 = Precipitation of the Wettest Month	0.04	0.26	-0.46
BIO14 = Precipitation of the Driest Month	0.23	-0.26	-0.05
BIO15 = Precipitation Seasonality (Coefficient of Variation)	-0.17	0.35	-0.08
BIO16 = Precipitation of the Wettest Quarter	0.03	0.26	-0.48
BIO17 = Precipitation of the Driest Quarter	0.24	-0.26	-0.06
BIO18 = Precipitation of the Warmest Quarter	-0.06	0.11	-0.33
BIO19 = Precipitation of the Coldest Quarter	0.21	-0.01	-0.21
Variance explained (%)	40.5	23.9	15.5
Cumulative variance explained (%)	40.5	64.4	79.9

Table 3. Associations of potential correlates of diversification with speciation rates. A weak significant correlation is apparent only between PC1 with DR

Variable	mean_rho	min_rho	max_rho	min_p	max_p
PC1	-0.103	-0.19283	-0.01606	0.053946	0.955045
PC2	0.01917	-0.08804	0.09211	0.377622	1.000999
PC3	-0.00166	-0.0822	0.066879	0.453546	1.000999
Elevation	0.04185	-0.04143	0.141316	0.177822	1.000999
Island	-0.01064	-0.10954	0.08603	0.301698	1.000999

Table 4. Summary of rates of climatic niche evolution in rhacophorid species occurring in different biogeographic regions.

(a) Model fit and estimated Brownian rate parameters for six regions (Africa, East/Southeast Asia, Himalayas, Peninsular India, Sundaland and Transitions) in the climatic niche axis of Rhacophoridae. The multiple-rate model of evolution provides the best fit to the data for these regions.

Trait	Single rate model		Multiple rate model		delta AICc	pchisq
	Likelihood	AICc	Likelihood	AICc		
PC1	-1113.51	2227.02	-1050.16	2100.33	126.69	0.04
PC2	-1058.21	2116.41	-1001.39	1902.78	213.63	0.00
PC3	-995.15	1990.31	-947.63	1695.27	295.04	0.00

(b) Model-averaged rate parameters for the measured six traits in the climatic niche axis of Rhacophoridae. There are intriguing differences in rates among traits in different biogeographic regions. The rates of evolution of PC1 and PC3 are significantly higher in species having a broad geographic distribution (Transitions).

Trait	Model averaged rates					
	Africa	East/ SE Asia	Himalaya	Peninsular India	Sundaland	Transitions
PC1	290.88	168.33	4186.69	155.17	475.48	2631.838
PC2	121.41	41.89	302.53	71.53	83.53	171.53
PC3	67.48	280.54	102.35	83.47	99.32	1957.35

Table 5. Summary of rates of climatic niche evolution in species of Rhacophoridae occurring on island and mainland regions.

(a) Model fit and estimated Brownian rate parameters for three traits (Mainland, Island and Island/Mainland) in the climatic niche axis of Rhacophoridae. The multiple-rate model of evolution provides the best fit to the data for these traits.

Trait	Single rate model		Multiple rate model		delta AICc	pchisq
	Likelihood	AICc	Likelihood	AICc		
PC1	-1113.51	2227.02	-1098.28	2196.57	30.46	0.08
PC2	-1058.21	2116.41	-1049.39	2098.78	17.63	0.10
PC3	-995.15	1990.31	-945.63	1891.27	99.04	0.00

(b) Model-averaged rate parameters for the measured three traits in the climatic niche axis of Rhacophoridae. There are intriguing differences in rates between traits in Islands and Mainland. Rates of evolution of PC1 and PC3 are significantly higher in species having a broad geographic distribution (Mainland/Island). Rates of climatic niche evolution in islands have increased ~ twofold relative to mainland species.

Trait	Model averaged rates		
	Island	Mainland	Mainland,Island
PC1	459.06	233.11	1248.53
PC2	304.41	210.89	340.53
PC3	67.48	277.54	1957.35

Table 6. Summary of rates of climatic niche evolution in different rhacophorid reproductive modes.

(a) Model fit and estimated Brownian rate parameters for four traits (AQ, GN, FN and DD) in the climatic niche axis of Rhacophoridae. The multiple-rate model of evolution provides the best fit to the data for these traits.

**Trait**	**Single rate model**		**Multiple rate model**		**delta AICc**	**pchisq**
	**Likelihood**	**AICc**	**Likelihood**	**AICc**		
PC1	-1113.51	2227.02	-1091.18	2162.57	64.45	0.00
PC2	-1058.21	2116.41	-1047.39	2083.78	32.63	0.00
PC3	-995.15	1990.31	-952.63	1959.27	31.04	0.00

(b) Model-averaged rate parameters for the measured reproductive modes in the climatic niche axis of Rhacophoridae. There are intriguing differences in rates among traits in different reproductive modes. Rates of evolution of DD and FN are significantly higher, which suggests that climatic niches have evolved at a significant rate in species having more terrestrial modes of reproduction.

**Trait**	**Model averaged rates**			
	**AQ**	**DD**	**FN**	**GN**
PC1	123.09	321.29	443.67	153.25
PC2	58.23	277.51	312.87	100.95
PC3	52.9	166.15	293.39	106.52

Table 7. GenBank accession numbers and voucher numbers of Anchor Hybrid Enrichment (AHE) data used in the current study.

Species	AHE voucher	GenBank Voucher number	GenBank Accession number						
			16S RNA	12S RNA	Rag-1	BDNF	Rhod	Cyt_B	Tyr
Beddomixalus bijui		TNHM (H) 12.6.18/57	KC594290	KC594289	KC594291	X	KC594292	X	X
Buergeria buergeri		KUHE 26541	AY880444	X	AB612031	X	X	AB529966	AB612033
Buergeria japonica		SCUM061101	X	X	GQ285754	GQ285691	GQ285783	KC151126	GQ285801
Chirixalus doriae	MVZ236721	FMNH255215	GQ204721	GQ204772	GQ204602	GQ204473	GQ204657	GQ204538	X
Chirixaluss nongkhorensis	CAS230907	FMNH255378	GQ204723	GQ204774	GQ204604	GQ204475	GQ204659	GQ204540	X
Chiromantis petersii		MVZ234168	GQ204733	GQ204784	X	X	X	GQ204550	X
Chiromantis rufescens	CAS207676	CAS	GQ204724	GQ204775	GQ204605	GQ204476	GQ204660	GQ204541	AY341748
Chiromantis xerampelina	ESP1249	MVZ234606	GQ204734	GQ204785	X	X	X	GQ204551	X
Feihyla fuhua		SCUM 0606132L	EU215546	X	X	X	X	X	X
Feihyla inexpectata		BORNENSIS 22421	AB813160	X	X	X	X	X	X
Feihyla kajau		ZMHAH518	KT382330	X	X	X	X	X	X
Feihyla palpebralis	FMNH254449	Vietnam712	GQ285681	GQ285681	GQ285772	GQ285709	GQ285792	X	GQ285810
Ghatixalus asterops		GA1602	KT359626	X	KT359636	X	X	X	X
Ghatixalus magnus		GM1604	KT359625	X	KT359635	X	X	X	X
Ghatixalus variabilis		SDB2010_275_F	KR259639	KR338959	KR534772	X	X	X	X
Gracixalus ananjevae		VNMO3012	JN862546	X	X	X	X	X	X
Gracixalus carinensis		ROM39660	GQ285670	GQ285670	GQ285762	GQ285699	GQ285788	X	GQ285806
Gracixalus gracilipes	AMNH_A163896	60821196	GQ285668	GQ285668	GQ285764	GQ285701	GQ285789	X	GQ285807
Gracixalus jinxiuensis	KIZ5287	KIZ 061210YP	EU215525	EU215525	GQ285763	GQ285700	EU215557	X	EU215587
Gracixalus nonggangensis		HMG200910010	JX841320	X	X	X	X	X	X
Gracixalus quangi		IEBR_A.2012.5	JX896683	X	X	X	X	X	X
Gracixalus quyeti		ZFMK_82999	EU871429	X	X	X	X	X	X

Gracixalus seesom		KUHE35088	LC011935	X	X	X	X	X	X
Gracixalus supercornutus	AMCC144966	AMS:R 173887	JN862545	X	X	X	X	X	X
Kurixalus appendiculatus		FMNH:267904	JQ060938	JQ060949	JQ060911	X	X	X	JQ060927
Kurixalus baliogaster	ROM33944	VNMN:03636	AB933301	X	X	X	X	X	X
Kurixalus banaensis		ROM32986	GQ285667	GQ285667	GQ285752	GQ285689	GQ285781	X	GQ285799
Kurixalus berylliniris		11311 (CE01X)	DQ468669	X	X	X	X	X	X
Kurixalus bisacculus	AMNH_A163974	MVZ:Herp:236725	JQ060929	JQ060940	KX554830	KX554903	X	X	JQ060916
Kurixalus chaseni		MZB_Amph_30594	MN727053	X	X	X	X	X	X
Kurixalus eiffingeri		UMFS 5969	DQ283122	DQ283122	X	X	DQ283830	X	DQ282931
Kurixalus gracilloides		ZMMUA6047	MN510866	X	X	X	X	X	X
Kurixalus hainanus		HNNU A1180	EU215548	EU215548	GQ285749	GQ285686	EU215578	X	X
Kurixalus idiootocus		ZRC1.1.5276	GQ204686	GQ204744	GQ204569	GQ204445	GQ204631	GQ204503	X
Kurixalus lenquanensis		YGH 20160143	KY768944	X	X	X	X	X	X
Kurixalus motokawai	AMNH_A163764	VNMN:03416	LC002886	X	X	X	X	X	X
Kurixalus naso		CAS 224469	DQ286973	X	X	X	X	X	X
Kurixalus odontotarsus		SCUM060688L	EU215549	EU215549	GQ285750	GQ285687	EU215579	X	X
Kurixalus verrucosus		CAS:231491	GU227333	GU227278	JQ060903	X	X	X	JQ060919
Kurixalus viridescens		VNMN_KHA004	AB933286	X	X	X	X	X	X
Kurixalus wangi		11328_CE06	DQ468671	X	X	X	X	X	X
Leptomantis angulirostris		UNIMAS 8681	AF215360	JN705322	X	KC961099	X	X	KC961221
Leptomantis cyanopunctatus	FMNH267837	NMBE 1056480	KC961084	KC961249	X	KC961098	X	X	KC961230
Leptomantis fasciatus		NMBE 1057405	KC961085	JN705330	X	KC961105	X	X	KC961225
Leptomantis gadingensis		NMBE 1057173	KC961087	KC961242	X	KC961102	X	X	KC961223
Leptomantis gauni		FMNH235047	GQ204714	GQ204765	GQ204596	X	GQ204650	GQ204531	X
Leptomantis harrissoni		NMBE 1056497	JN377359	JN705332	X	KC961107	X	X	KC961227
Leptomantis penanorum		ZMH A10168	JN377349	JN705323	X	KC961100	X	X	KC961222

Leptomantis robinsonii		LSUHC_5681	MH590207	X	X	X	X	X	X
Leptomantis rufipes		NMBE 1057529	KC961086	JN705333	X	KC961108	X	X	KC961229
Liuixalus calcarius		NAPCHN03587	KT198741	X	X	X	X	X	X
Liuixalus feii		SYSa0002390	KT198734	X	X	X	X	X	X
Liuixalus hainanus		060401L	GQ285671	GQ285671	GQ285757	GQ285694	GQ285785	X	GQ285803
Liuixalus ocellatus		HN0806045	GQ285672	GQ285672	GQ285755	GQ285692	GQ285784	X	GQ285802
Liuixalus romeri		KIZ060821245	EF564535	EF564463	EU924514	X	EU924542	EU924598	EU924570
Liuixalus shiwandashan		2015CIB102438	KT192633	X	X	X	X	X	X
Mercurana myristicapalustris		TNHM (H) 12.6.18/74	KC594294	KC594293	KC594295	X	KC594296	X	X
Nasutixalus jerdonii		SL48	MF319221	X	X	X	X	X	X
Nasutixalus medogensis	KIZ016395	KIZ016395	KU243082	KU243082	X	X	X	X	X
Nasutixalus yingjiangensis		SYSa005804	MG603590	MG603587	X	X	X	X	X
Nyctixalus margaritifer		KUHE26135	LC012864	X	X	X	X	X	X
Nyctixalus pictus	MVZ239498	MVZ239460	GQ204732	GQ204783	GQ204613	GQ204483	GQ204666	GQ204549	X
Nyctixalus spinosus		Nsp1	KT461916	X	X	X	X	X	X
Philautus acutirostris		RMB 589	AY326059	X	X	X	X	X	X
Philautus acutus		NMBE 1056431	JN705366	JN705337	X	KC961117	X	X	KC961191
Philautus amabilis		UTA-A_63816	KY435420	X	X	X	X	X	X
Philautus amoenus	FMNH252417	X	X	X	X	X	X	X	X
Philautus aurantium		FMNH233226	GQ204705	GQ204756	GQ204587	GQ204460	GQ204642	GQ204522	X
Philautus aurifasciatus		ZRC1.1.5267/1.5267?	GQ204702	AY141805	GQ204584	GQ204458	GQ204640	GQ204519	X
Philautus bunitus		UNIMAS 9045	JN705368	JN705368	X	KC961120	X	X	KC961194
Philautus cornutus		MZB.Amph.26178	KY435421	X	X	X	X	X	X
Philautus davidlabangi		ZMH A10429	JN705387	JN705357	X	X	X	X	X
Philautus disgregus		FMNH231141	GQ204704	GQ204755	GQ204586	GQ204459	GQ204641	GQ204521	X
Philautus everetti		KU309610	JN705377	X	X	X	X	X	X

Philautus hosii	FMNH273835	NMBE 1057287	JN705384	JN705353	X	KC961113	X	X	KC961205
Philautus ingeri	FMNH239280	FMNH239280	GQ204706	GQ204757	GQ204588	GQ204461	X	GQ204523	X
Philautus kakipanjang		BH82	KX440526	X	X	X	X	X	X
Philautus kerangae		MZB.Amph.26739	KY435423	X	X	X	X	X	X
Philautus larutensis		LSUHC10839	MH590213	X	X	X	X	X	X
Philautus macroscelis		NMBE 1056486	JN705375	JN705346	X	KC961112	X	X	KC961202
Philautus mjobergi		FMNH252411	GQ204708	GQ204759	GQ204590	GQ204463	GQ204644	GQ204525	X
Philautus nephophilus		BORN 22666	KT445971	X	X	X	X	X	X
Philautus nianeae		NCSM 80926	KF723233	X	X	X	X	X	X
Philautus petersi		NMBE 1056443	JN705381	JN705350	X	KC961134	X	X	KC961204
Philautus polymorphus		UTA-A_63940	KY435432	X	X	X	X	X	X
Philautus refugii		ZMH A10415	JN705383	JN705352	X	KC961136	X	X	KC961201
Philautus surdus		FMNH 259008	DQ286991	X	X	X	X	X	X
Philautus tectus		NMBE 1056451	JN705370	JN705341	X	KC961115	X	X	KC961208
Philautus umbra		NMBE 1056454	JN705379	JN705348	X	KC961133	X	X	KC961198
Philautus ventrimaculatus		UTA-A_63868	KY435429	X	X	X	X	X	X
Philautus vermiculatus		LSUHC10753	MH590210	X	X	X	X	X	X
Philautus worcesteri	FMNH250626	FMNH250626	GQ204707	GQ204758	GQ204589	GQ204462	GQ204643	GQ204524	X
Polypedates braueri		KUHE:37040	AB728016	X	AB728252	AB728194	AB728274	X	AB728308
Polypedates colletti		FMNH242765	GQ204697	GQ204750	GQ204579	GQ204454	X	X	X
Polypedates cruciger		WHT2640	GQ204687	AY141799	GQ204570	GQ204446	GQ204632	GQ204504	X
Polypedates discantus		ZMKU_AM_00990	KF303477	X	X	X	X	X	X
Polypedates impresus		LC0805113	KF053209	X	X	X	X	X	X
Polypedates leucomystax	CAS241415	FMNH255296	GQ204700	GQ204753	GQ204582	GQ204457	X	GQ204517	X
Polypedates macrotis	CAS229555	FMNH239119	GQ204695	GQ204748	GQ204577	GQ204452	GQ204638	GQ204512	X
Polypedates maculatus	CAS233186	WHTKANT	GQ204694	X	GQ204576	GQ204451	GQ204637	GQ204511	X

Polypedates megacephalus		KUHE:16589	AB728085	X	AB728258	AB728200	AB728278	X	AB728312
Polypedates mutus	AMNH_A168760	SCUM37940C	EU215551	EU215551	GQ285770	GQ285707	EU215581	X	EU215611
Polypedates otilophus		FMNH239147	GQ204696	GQ204749	GQ204578	GQ204453	GQ204639	GQ204513	X
Polypedates teraiensis		Pter_Bd9	AB530520	X	X	X	X	X	X
Polypedates bengalensis		KM2	MK490922	X	X	X	X	X	X
Pseudohphilautus. cf. folicola2		WHT2531	MH789427	X	MK007006	X	X	X	X
Pseudophilautus abundus		WHT3231	MH789433	MK020187	MK007029	X	X	X	X
Pseudophilautus alto	R1332	WHT5029	GQ204677	GQ204738	GQ204562	GQ204440	X	GQ204494	X
Pseudophilautus asankai		WHT5107	FJ788160	FJ788141	X	X	X	X	X
Pseudophilautus auratus		WHT2887	FJ788164	FJ788145	X	X	X	X	X
Pseudophilautus caeruleus		WHT2511	AY141810	AY141764	MK007003	X	X	X	X
Pseudophilautus cavirostris		WHT3299	GQ204676	GQ204737	GQ204561	GQ204439	GQ204622	GQ204493	X
Pseudophilautus cf. mooreorum		WHT6306	MH789446	MK020197	MK007042	X	X	X	X
Pseudophilautus cuspis		WHT5974	MH789438	MK020192	MK007035	X	X	X	X
Pseudophilautus decoris		WHT3271	FJ788163	FJ788144	MK007030	X	X	X	X
Pseudophilautus dilmah		HFS006	KP272046	X	X	X	X	X	X
Pseudophilautus femoralis		WHT2772	AY141831	AY141785	MK007016	X	X	X	X
Pseudophilautus folicola		WHT6114	GQ204680	X	GQ204564	X	GQ204625	GQ204497	X
Pseudophilautus frankenbergi		WHT2552	AY141814	AY141768	MK007008	X	X	X	X
Pseudophilautus fulvus		WHT3121	MH789430	MK020184	MK007020	X	X	X	X
Pseudophilautus hallidayi		WHT2886	MH789429	MK020183	MK007019	X	X	X	X
Pseudophilautus hankeni		WHT6302	MH789444	MK020195	MK007040	X	X	X	X
Pseudophilautus hoffmanni		WHT3223	GQ204673	GQ204736	GQ204558	GQ204436	GQ204619	GQ204490	X
Pseudophilautus hoipolloi		WHT2675	AY141822	AY141776	MK007012	X	X	X	X
Pseudophilautus limbus		WHT2700	GQ204668	AY141779	GQ204553	X	X	GQ204485	X
Pseudophilautus lunatus		WHT3283	GQ204675	FJ788150	GQ204560	GQ204438	GQ204621	GQ204492	X

Pseudophilautus macropus		WHT2484	AY141808	AY141762	X	X	X	X	X
Pseudophilautus microtympanum	R1284	WHT5065	GQ204678	GQ204739	GQ204563	GQ204441	GQ204623	GQ204495	AF249189
Pseudophilautus mooreorum		WHT3209	FJ788153	FJ788134	MK007025	X	X	X	X
Pseudophilautus ocularis		WHT2792	AY141835	AY141789	MK007018	X	X	X	X
Pseudophilautus papillosus		WHT3284	FJ788170	FJ788151	MK007031	X	X	X	X
Pseudophilautus pleurotaenia		WHT3176	FJ788165	FJ788146	MK007021	X	X	X	X
Pseudophilautus poppiae		WHT2779	GQ204670	FJ788136	GQ204555	X	GQ204616	GQ204487	X
Pseudophilautus popularis		WHT3191	FJ788168	FJ788149	MK007023	X	X	X	X
Pseudophilautus procax		WHT2786	AY141834	AY141788	MK007017	X	X	X	X
Pseudophilautus regius		WHT3515	GQ204682	GQ204742	GQ204566	X	GQ204627	X	X
Pseudophilautus reticulatus		WHT3230	GQ204674	X	GQ204559	GQ204437	GQ204620	GQ204491	X
Pseudophilautus rus		WHT5871	MH789437	MK020191	MK007034	X	X	X	X
Pseudophilautus sarasinorum		WHT2481	GQ204667	AY141761	GQ204552	X	GQ204614	GQ204484	X
Pseudophilautus schmarda		WHT2715	GQ204669	AY141780	GQ204554	GQ204435	GQ204615	GQ204486	X
Pseudophilautus schneideri		WHT2667	AY141820	AY141774	MK007010	X	X	X	X
Pseudophilautus semiruber		WHT5831	MH789436	MK020190	MK007033	X	X	X	X
Pseudophilautus silus		WHT6313	MH789447	MK020198	MK007043	X	X	X	X
Pseudophilautus silvaticus		WHT2515	AY141811	AY141765	MK007004	X	X	X	X
Pseudophilautus simba		WHT6004	GQ204679	GQ204740	X	GQ204442	GQ204624	GQ204496	X
Pseudophilautus singu		WHT6034	MH789440	X	MK007037	X	X	X	X
Pseudophilautus sordidus		WHT2699	AY141824	AY141778	X	X	X	X	X
Pseudophilautus steineri		WHT3210	FJ788157	FJ788138	MK007026	X	X	X	X
Pseudophilautus stellatus		HFS01002	JN862536	JN862535	X	X	X	X	X
Pseudophilautus stictomerus		WHT3301	MH789434	MK020188	MK007032	X	X	X	X
Pseudophilautus stuarti		WHT3207	GQ204672	GQ204735	GQ204557	X	GQ204618	GQ204489	X
Pseudophilautus tanu		WHT6343	FJ788171	FJ788152	X	X	X	X	X

Pseudophilautus viridis		WHT2766	AY141830	AY141784	MK007015	X	X	X	X
Pseudophilautus wynaadensis		GQ204685	GQ204685	GQ204743	GQ204568	X	GQ204630	GQ204502	AF249190
Pseudophilautus zorro		WHT3175	GQ204671	FJ788147	GQ204556	X	GQ204617	GQ204488	X
Pseudophilautus. cf. folicola1		WHT2525	AY141812	AY141766	MK007005	X	X	X	X
Pseudophilautus. cf. frankenbergi		WHT2729	AY141828	AY141782	MK007014	X	X	X	X
Pseudophilautus. cf. popularis		WHT6010	MH789439	MK020193	MK007036	X	X	X	X
Raorchestes agasthyaensis		CESF492	JX092723	X	X	X	JX092980	JX092785	JX092926
Raorchestes akroparallagi		CESF061	JX092650	JX092726	X	X	JX092981	JX092786	JX092927
Raorchestes andersoni	KIZYPX16167	X	X	X	X	X	X	X	X
Raorchestes aureus		CESF1164	KM596540	X	X	X	X	X	X
Raorchestes bobingeri		CESF1238	JX092680	JX092733	X	X	X	JX092795	JX092930
Raorchestes charius		CESF132	JX092691	JX092736	X	X	JX092989	JX092799	JX092933
Raorchestes chlorosomma		FB_2008c	EU450017	X	X	X	X	X	X
Raorchestes chromasynchysi		CESF1127	JX092667	JX092738	X	X	JX092990	JX092802	JX092935
Raorchestes crustai		CESF1199	JX092677	JX092742	X	X	X	JX092806	JX092940
Raorchestes dubois		CESF114	JX092668	X	X	X	JX092993	JX092808	JX092942
Raorchestes flaviocularis		CESF1252	KM596549	X	X	X	X	X	X
Raorchestes ghatei	R1267	AGCZRL	KF366391	X	X	X	X	X	X
Raorchestes graminirupes		CESF044	JX092649	JX092772	X	X	X	JX092812	JX092946
Raorchestes gryllus	ROM30298	ROM30288	GQ285674	GQ285674	GQ285777	GQ285714	GQ285796	X	GQ285814
Raorchestes indigo		CESF138	KM596557	X	X	X	X	X	X
Raorchestes jayarami		CESF1260	JX092686	JX092750	X	X	JX093000	JX092816	JX092948
Raorchestes johnceei		CESF1236	JX092679	JX092751	X	X	JX093001	JX092817	JX092949
Raorchestes kakachi		CESF1385	KM596558	X	X	X	X	X	X
Raorchestes luteolus		CESF1012	JX092659	JX092756	X	X	JX093004	JX092823	JX092954
Raorchestes manohari		CESF1187	JX092674	X	X	X	JX093005	JX092824	JX092955

Raorchestes menglaensis		060821286Rao	GQ285676	GQ285676	GQ285778	GQ285715	GQ285797	X	GQ285815
Raorchestes munnarensis		CESF094	JX092655	X	X	X	JX093008	JX092828	JX092959
Raorchestes parvulus	CAS233160	LSUHC11118	MH590201	X	X	X	X	X	X
Raorchestes ponmudi		CESF063	JX092651	JX092762	X	X	JX093011	JX092832	JX092963
Raorchestes primarrumpfi		CESF442	KM596575	X	X	X	X	X	X
Raorchestes resplendens		CESF1258	JX092683	X	X	X	JX093013	JX092835	JX092965
Raorchestes signatus	R1265	Genbank	GQ204684	AY141795	GQ204567	GQ204444	GQ204629	JX092836	JX092966
Raorchestes sushili		CESF1259	JX092684	JX092766	X	X	JX093018	JX092844	JX092972
Raorchestes theuerkaufi		CESF1342	JX092693	JX092767	X	X	X	JX092845	X
Raorchestes travancoricus		CESF473	JX092721	JX092776	X	X	JX093019	JX092847	JX092974
Raorchestes uthamani		CESF483	JX092722	X	X	X	JX093020	JX092849	X
Raorchestes archeos		CESF1190	JX092675	X	X	X	X	X	X
Raorchestes blandus		CESF104	JX092660	X	X	X	X	X	X
Raorchestes cangyuanensis		KIZ015855	MN475866	X	X	X	X	X	X
Raorchestes echinatus		CESF1414	JX092696	X	X	X	X	X	X
Raorchestes hassanensis		CESF1178	JX092673	X	X	X	X	X	X
Raorchestes honnametti		5941	KT151650	X	X	X	X	X	X
Raorchestes lechiya		RL1601	KT359622	X	X	X	X	X	X
Raorchestes leucolatus		CESF1147	JX092669	X	X	X	X	X	X
Raorchestes rezakhani		A0319	MN072374	X	X	X	X	X	X
Raorchestes sanctisilvaticus		CESF1274	JX092689	X	X	X	X	X	X
Raorchestes shillongensis		CESF418	JX092711	X	X	X	X	X	X
Raorchestes silentvalley		RS1601	KT359628	X	X	X	X	X	X
Rhacophorus annamensis	AMNH_A163747	FMNH253934	GQ204717	GQ204768	GQ204598	GQ204470	GQ204653	GQ204534	X
Rhacophorus bengkuluensis		UTA_A_62770	KM212948	X	X	X	X	X	X
Rhacophorus bipunctatus		FMNH253114	GQ204716	GQ204767	X	GQ204469	GQ204652	GQ204533	X

Rhacophorus borneensis		BORN_22411	AB781694	X	X	X	X	X	X
Rhacophorus calcaneus		FMNH256465	GQ204719	GQ204770	GQ204600	X	GQ204655	GQ204536	X
Rhacophorus catamitus		ENS_7610	JF748392	X	X	X	X	X	X
Rhacophorus edentulus		MZB:Amp:26628	MH751450	X	X	X	X	X	X
Rhacophorus exechopygus	AMCC192785	RH06036	GQ469976	X	X	X	X	X	X
Rhacophorus georgii		MZB:Amp:23395	MH751453	X	X	X	X	X	X
Rhacophorus helenae		ABV00238	KX139178	X	X	X	X	X	X
Rhacophorus hoabinhensis		TAO	LC331096	X	X	X	X	X	X
Rhacophorus hoanglienensis	AMNH_A163767	X	X	X	X	X	X	X	X
Rhacophorus indonesiensis		AH-2014b	AB983368	X	X	X	X	X	X
Rhacophorus kio	KIZYN080283	SCUM 37941C	EU215532	EU215532	GQ285766	GQ285703	EU215562	X	EU215592.
Rhacophorus lateralis		Rlat_In	AB530548	X	X	X	X	X	X
Rhacophorus malabaricus		Genbank	AF249050	AF249029	X	X	AF249125	AF249094	AF249188
Rhacophorus margaritifer		ENS_15850	KX398887	X	X	X	X	X	X
Rhacophorus modestus		ENS16851	KX398903	X	X	X	X	X	X
Rhacophorus monticola		RMB_1236	AY326060	X	X	X	X	X	X
Rhacophorus nigropalmatus	FMNH230905	FMNH230902	GQ204710	GQ204761	GQ204592	GQ204465	GQ204646	GQ204527	X
Rhacophorus norhayatii		NNRn	AB728191	X	AB728270	AB728214	AB728287	X	AB728321
Rhacophorus orlovi	AMNH_A161407	LJT R44	KC465840	X	X	X	X	X	X
Rhacophorus poecilonotus		ENS14103	KX398909	X	X	X	X	X	X
Rhacophorus pseudomalabaricus		SDB_2011_1010	KC593855	X	X	X	X	X	X
Rhacophorus reinwardtii		FMNH235034	GQ204713	GQ204764	GQ204595	GQ204468	GQ204649	GQ204530	X
Rhacophorus rhodopus	CAS224677	KIZ060821037	EF564572	EF564500	EU924532	X	EU924560	EU924616	EU924588
Rhacophorus robertingeri		VNMN3446	LC010615	X	X	X	X	X	X
Rhacophorus spelaeus		Genbank	LC331095	X	X	X	X	X	X
Rhacophorus translineatus	KIZ6648	Rao6237	JX219449	X	X	X	X	X	X

Rhacophorus vampyrus		AMS_R_173132	HQ656819	X	X	X	X	X	X
Rhacophorus verrucopus		Rao6254	JX219436	X	X	X	X	X	X
Rhacophorus calcadensis		SDB.2011.291	KC571276	X	X	X	X	X	X
Rohanixalus hansenae	KIZ9387	0046Y	KR827734	X	X	X	X	X	X
Rohanixalus vittatus		KIZ 0001Rao	GQ285684	GQ285684	GQ285774	GQ285711	X	X	GQ285811
Taruga eques		WHT2741	AY141847	AY141801	GQ204571	GQ204447	GQ204633	GQ204505	X
Taruga fastigo		WHT2783	AY141848	AY141802	GQ204572	GQ204448	GQ204634	GQ204506	X
Taruga longinasus		WHTKAN1	GQ204691	GQ204745	GQ204573	GQ204449	GQ204635	GQ204507	X
Theloderma albopunctatum		KIZ060821217	EF564522	X	X	X	X	X	X
Theloderma annae		IEBR3734	LC168172	X	X	X	X	X	X
Theloderma asperum		KIZ060821201	EF564521	EF564449	EU924534	X	EU924562	EU924618	EU924590
Theloderma auratum		ZMMU_NAP064022	MG917772	X	X	X	X	X	X
Theloderma baibungense		KIZYPX37270	KU243080	X	X	X	X	X	X
Theloderma bicolor		VNMN:1394	JX046475	KF991263	KF991342	KF991305	X	KF991324	X
Theloderma corticale	MVZ225131	AMNH A161499	X	DQ283050	X	X	DQ283779	X	DQ282904
Theloderma gordoni	ROM30263	VNMN:4407	LC012852	X	X	X	X	X	X
Theloderma horridum		LSUHC_8342	MH590197	X	X	X	X	X	X
Theloderma lacustrinum		NCSM84683	KX095246	X	X	X	X	X	X
Theloderma laeve		NAP01644	KT461907	X	X	X	X	X	X
Theloderma lateriticum		VNMN_1216	LC012851	X	X	X	X	X	X
Theloderma leporosum		Tlep1	KT461922	X	X	X	X	X	X
Theloderma licin		KUHE19426	LC012859	X	X	X	X	X	X
Theloderma moloch	KIZ029453	KIZYPX31941	KU243081	X	X	X	X	X	X
Theloderma nebulosum		VNMNROM_39588	LC012845	X	X	X	X	X	X
Theloderma palliatum		NAP01846	KT461901	KT461893	X	X	X	X	X
Theloderma petilum	KIZ24310	HNUE MNA.2012.0001	KJ802925	X	X	X	X	X	X

Theloderma phrynoderma		CAS:247910	KJ128283	KJ128281	X	KU244404	KJ128279	X	KJ128277
Theloderma pyaukkya		CAS 234857	KU244371	X	X	X	X	X	X
Theloderma rhododiscus		KIZ060821063	EF564533	EF564461	EU924535	X	EU924563	EU924619	EU924591
Theloderma ryabovi		Try1	KT461914	X	X	X	X	X	X
Theloderma stellatum		NAP03961	KT461917	X	X	X	X	X	X
Theloderma truongsonense	AMNH_A191915	ROM-39363	KT461925	X	X	X	X	X	X
Theloderma vietnamense	KIZ10652	NCSM 80384	KU561887	X	X	KU561888	KU561891	X	KU561897
Zhangixalus achantharrhena		ENS 15995	KX398872	X	X	X	X	X	X
Zhangixalus arboreus		KUHE 24248	AY880523	AY880610	X	X	AY880653	X	X
Zhangixalus burmanus	KIZYPX16626	SCUM 060614L	EU215537	X	EU924533	X	EU215567	EU924617	EU215597
Zhangixalus chenfui	KIZ23210	FMNH232964	GQ204712	GQ204763	GQ204594	GQ204467	GQ204648	GQ204529	X
Zhangixalus dorsoviridis	KIZ9135	ROM38011	JX219427	X	X	X	X	X	X
Zhangixalus duboisi		YGH080158	EU924624	EU924629	EU924530	X	EU924558	EU924614	EU924586
Zhangixalus dugritei		SCUM 051001L	EU215541	EU215541	GQ285768	GQ285705	EU215571	EU215601	X
Zhangixalus dulitensis	FMNH240957	FMNH235741	GQ204715	GQ204766	GQ204597	X	GQ204651	GQ204532	X
Zhangixalus feae	FMNH257914	KIZ060821197	EF564546	EF564474	EU924522	X	EU924550	EU924606	EU924578
Zhangixalus hongchibaensis		CIB 097687	JN688883	X	X	X	JN688897	X	JN688906
Zhangixalus hui		KIZ07052101	EU924622	EU924627	EU924523	X	EU924551	EU924607	EU924579
Zhangixalus hungfuensis		SCUM 060425L	EU215538	X	X	X	EU215568	X	EU215598
Zhangixalus lishuiensis		YPX47792	KY653720	X	X	X	X	X	X
Zhangixalus minimus		KIZ060821020	EF564561	EF564489	EU924525	X	EU924553	EU924609	EU924581
Zhangixalus moltrechti		SCUM 061106L	EU215543	X	X	X	EU215573	X	EU215603
Zhangixalus nigropunctatus	GZ070667	KIZ07061001	EU924623	EU924628	EU924527	X	EU924555	EU924611	EU924583
Zhangixalus omeimontis	KIZYPX20358	KIZ060821282	EF564564	EF564492	EU924528	X	EU924556	EU924612	EU924584
Zhangixalus pachyproctus		VNMN:1299	LC545592	X	X	X	X	X	X
Zhangixalus pinglongensis		NHMGCHN201002011	KU170684	X	X	X	X	X	X

Zhangixalus prominanus		ENS14604	KX398928	X	X	X	X	X	X
Zhangixalus puerensis	KIZYN0705184	SCUM 060649L	EU215542	X	X	X	EU215572	X	EU215602
Zhangixalus schlegelii		KUHE 26251	AY880528	AY880615	X	X	AY880658	X	X
Zhangixalus smaragdinus	CAS235817	KIZ060821140	EF564548	EF564476	EU924524	X	EU924552	EU924608	EU924580
Zhangixalus viridis		KUHE:35354	LC386576	X	X	X	X	X	X
Zhangixalus wui		CIB 097685	JN688881	X	X	X	JN688896	X	JN688910
Zhangixalus zhoukaiye		AHU-RhaDb-150428	KU601502	X	X	X	X	X	X

Table 8. References used to establish phylogenetic relationships among rhacophorid species whose genetic data are lacking. These studies were useful in imposing constraints for 74 species on the phylogenetic tree. A total of 94 species lack genetic data. The remaining 20 species were not constrained, as their original descriptions do not provide sufficient information to determine their relative position in the phylogeny.

Species	Reference
Buergeria otai	Wang, Y.-H., Y.-W. Hsiao, K.-H. Lee, H.-Y. Tseng, Y.-P. Lin, S. Komaki, and S.-M. Lin. 2018 "2017". Acoustic differentiation and behavioral response reveals cryptic species within *Buergeria* treefrogs (Anura, Rhacophoridae) from Taiwan. *PLoS One* 12(9: e0184005): 1–24.
Buergeria robusta	Wang, Y.-H., Y.-W. Hsiao, K.-H. Lee, H.-Y. Tseng, Y.-P. Lin, S. Komaki, and S.-M. Lin. 2018 "2017". Acoustic differentiation and behavioral response reveals cryptic species within *Buergeria* treefrogs (Anura, Rhacophoridae) from Taiwan. *PLoS One* 12(9: e0184005): 1–24.
Chirixalus baladika	Riyanto, A., and H. Kurniati. 2014. Three new species of *Chiromantis* Peters 1854 (Anura: Rhacophoridae) from Indonesia. *Russian Journal of Herpetology* 21: 65–73.
Chirixalus trilaksonoi	Riyanto, A., and H. Kurniati. 2014. Three new species of *Chiromantis* Peters 1854 (Anura: Rhacophoridae) from Indonesia. *Russian Journal of Herpetology* 21: 65–73.
Chirixalus dudhwaensis	Wilkinson, J.A., Win, H., Thin, T., Lwin, K.S., Shein, A.K., & Tun, H.M. (2003). A new species of *Chirixalus* (Anura: Rhacophoridae) from western Myanmar (Burma). *Proceedings of the California Academy of Sciences, 4th series*. 54:17-26
Chirixalus shyamirupus	Wilkinson, J.A., Win, H., Thin, T., Lwin, K.S., Shein, A.K., & Tun, H.M. (2003). A new species of *Chirixalus* (Anura: Rhacophoridae) from western Myanmar (Burma). *Proceedings of the California Academy of Sciences, 4th series*. 54:17-26
Chirixalus simus	Wilkinson, J.A., Win, H., Thin, T., Lwin, K.S., Shein, A.K., & Tun, H.M. (2003). A new species of *Chirixalus* (Anura: Rhacophoridae) from western Myanmar (Burma). *Proceedings of the California Academy of Sciences, 4th series*. 54:17-26
Chirixalus punctatus	Wilkinson, J.A., Win, H., Thin, T., Lwin, K.S., Shein, A.K., & Tun, H.M. (2003). A new species of *Chirixalus* (Anura: Rhacophoridae) from western Myanmar (Burma). *Proceedings of the California Academy of Sciences, 4th series*. 54:17-26
Chiromantis kelleri	Poynton, J.C. (2000). Foam-nest treefrogs in eastern Africa (Anura Rhacophoridae *Chiromantis*): Taxonomic complexities, *African Journal of Herpetology* 49(2): 111-128. DOI: 10.1080/21564574.2000.9635438
Chirixalus senapatiensis	Mathew, R., and N. Sen. (2009). Studies on little known amphibians of Northeast India. *Records of the Zoological Survey of India. Occasional Papers* 293(23): 1–64.
Feihyla fuhua	Fei, L., Ye, C. Y and Jiang, J. P. (2010). A new species of Rhacophoridae

	from Yunnan, China (Amphibia, Anura). *Acta Zootaxonomica Sinica* 35: 413–417
Feihyla samkonensis	Biju et al. (2020). New insights on the systematics and reproductive behaviour in tree frogs of the genus *Feihyla*, with description of a new related genus from Asia (Anura, Rhacophoridae). *Zootaxa* 4878, 1–55
Feihyla vittiger	Biju et al. (2020). New insights on the systematics and reproductive behaviour in tree frogs of the genus *Feihyla*, with description of a new related genus from Asia (Anura, Rhacophoridae). *Zootaxa* 4878, 1–55
Gracixalus guangdongensis	Wang, J., Zeng, Z. C., Liu, Z. Y. and Wang, Y. Y. (2018). Description of a new species of *Gracixalus* (Amphibia: Anura: Rhacophoridae) from Guangdong Province, southeastern China. *Zootaxa* 4420: 251–269.
Gracixalus jinggangensis	Wang, J., Zeng, Z. C., Liu, Z. Y. and Wang, Y. Y. (2018). Description of a new species of *Gracixalus* (Amphibia: Anura: Rhacophoridae) from Guangdong Province, southeastern China. *Zootaxa* 4420: 251–269.
Gracixalus lumarius	Wang, J., Zeng, Z. C., Liu, Z. Y. and Wang, Y. Y. (2018). Description of a new species of *Gracixalus* (Amphibia: Anura: Rhacophoridae) from Guangdong Province, southeastern China. *Zootaxa* 4420: 251–269.
Gracixalus medogensis	Ye, C.Y., and Hu, S. Q. (1984). A new species of *Philautus* (Anura: Rhacophoridae) from Xizang Autonomous Region. *Acta Herpetologica Sinica* 3(4): 67–69.
Gracixalus tianlinensis	Chen, W.C., Bei, Y. J., Liao, X., Zhou, S. C and Mo, Y. M (2018). A new species of *Gracixalus* (Anura: Rhacophoridae) from West Guangxi, China. *Asian Herpetological Research* 9: 74–84.
Gracixalus trieng	Rowley, J. J. L., Le, D. T. T., Hoang, H. D., Cao, T. T. and Dau, V. Q. (2020). A new species of phytotelm breeding frog (Anura: Rhacophoridae) from the Central Highlands of Vietnam. *Zootaxa* 4779: 341–354 (https://doi.org/10.11646/zootaxa.4779.3.3).
Gracixalus yunnanensis	Rowley, J. J. L., Le, D. T. T., Hoang, H. D., Cao, T. T. and Dau, V. Q. (2020). A new species of phytotelm breeding frog (Anura: Rhacophoridae) from the Central Highlands of Vietnam. *Zootaxa* 4779: 341–354 (https://doi.org/10.11646/zootaxa.4779.3.3).
Kurixalus absconditus	Mediyansyah, A. Hamidy, M. Munir, and M. Matsui. (2019). A new tree frog of the genus *Kurixalus* Ye, Fei & Dubois, 1999 (Amphibia: Rhacophoridae) from West Kalimantan, Indonesia. Treubia. *Batavia* 46: 51–72 (DOI:10.14203/treubia.v46i0.3790).
Kurixalus yangi	Yu, G., Hui, H., Rao, D. Q and Yang, J. X. (2018). A new species of *Kurixalus* from western Yunnan, China (Anura, Rhacophoridae). *ZooKeys* 770: 211–226.
Kurixalus chaseni	Mediyansyah, A. Hamidy, M. Munir, and M. Matsui. (2019). A new tree frog of the genus *Kurixalus* Ye, Fei & Dubois, 1999 (Amphibia: Rhacophoridae) from West Kalimantan, Indonesia. Treubia. *Batavia* 46: 51–72 (DOI:10.14203/treubia.v46i0.3790).
Kurixalus gracilloides	Nguyen, T. V., Duong, T. V., Luu, K. T and Poyarkov, Jr., N. A. (2020). A

	new species of *Kurixalus* (Anura: Rhacophoridae) from northern Vietnam with comments on the biogeography of the genus. *Journal of Natural History London* 54: 195–223 (https://doi.org/10.1080/00222933.2020.1728411).
Kurixalus lenquanensis	Nguyen, T. V., Duong, T. V., Luu, K. T and Poyarkov, Jr., N. A. (2020). A new species of *Kurixalus* (Anura: Rhacophoridae) from northern Vietnam with comments on the biogeography of the genus. *Journal of Natural History London* 54: 195–223 (https://doi.org/10.1080/00222933.2020.1728411).
Leptomantis bimaculatus	Gonzalez, P., Su, Y. C., Siler, C. D., Barley, A. J., Sanguila, M. B., Diesmos, A. C. and Brown, R. M. (2014). Archipelago colonization by ecologically dissimilar amphibians: Evaluating the expectation of common evolutionary history of geographical diffusion in co-distributed rainforest tree frogs in islands of Southeast Asia. *Molecular Phylogenetics and Evolution* 72: 35–41.
Leptomantis malkmusi	Dehling, J. M. (2015). A new species of *Rhacophorus* (Anura: Rhacophoridae) from Gunung Kinabalu, Borneo. *Salamandra* 51: 1–11.
Leptomantis pseudacutirostris	Dehling, J. M. (2011). Taxonomic status of the population of *Rhacophorus angulirostris* Ahl, 1927 (Anura: Rhacophoridae) from Sumatera Barat (West Sumatra) and its description as a new species. *Salamandra* 47: 133–143.
Philautus acutirostris	Wostl, E., Riyanto, A., Hamidy, A., Kurniawan, N., Smith, E. N. and Harvey, M. B. (2017). A taxonomic revision of the *Philautus* (Anura: Rhacophoridae) of Sumatra with the description of four new species. *Herpetological Monographs* 31: 98–141.
Philautus amabilis	Wostl, E., Riyanto, A., Hamidy, A., Kurniawan, N., Smith, E. N. and Harvey, M. B. (2017). A taxonomic revision of the *Philautus* (Anura: Rhacophoridae) of Sumatra with the description of four new species. *Herpetological Monographs* 31: 98–141.
Philautus polymorphus	Wostl, E., Riyanto, A., Hamidy, A., Kurniawan, N., Smith, E. N. and Harvey, M. B. (2017). A taxonomic revision of the *Philautus* (Anura: Rhacophoridae) of Sumatra with the description of four new species. *Herpetological Monographs* 31: 98–141.
Philautus thamyridion	Wostl, E., Riyanto, A., Hamidy, A., Kurniawan, N., Smith, E. N. and Harvey, M. B. (2017). A taxonomic revision of the *Philautus* (Anura: Rhacophoridae) of Sumatra with the description of four new species. *Herpetological Monographs* 31: 98–141.
Philautus ventrimaculatus	Wostl, E., Riyanto, A., Hamidy, A., Kurniawan, N., Smith, E. N. and Harvey, M. B. (2017). A taxonomic revision of the *Philautus* (Anura: Rhacophoridae) of Sumatra with the description of four new species. *Herpetological Monographs* 31: 98–141.
Philautus cornutus	Wostl, E., Riyanto, A., Hamidy, A., Kurniawan, N., Smith, E. N. and Harvey, M. B. (2017). A taxonomic revision of the *Philautus* (Anura: Rhacophoridae) of Sumatra with the description of four new species. *Herpetological Monographs* 31: 98–141.
Philautus erythrophthalmus	Stuebing, R. B., and Wong, A. (2000). A new species of frog, *Philautus erythrophthalmus* (Rhacophoridae) from southwestern Sabah, Malaysia.

	Raffles Bulletin of Zoology. Singapore 48: 293–296.
Philautus gunungensis	Bossuyt, F., and Dubois, A. (2001). A review of the frog genus *Philautus* Gistel, 1848 (Amphibia, Anura, Ranidae, Rhacophorinae). *Zeylanica* 6: 1–112.
Philautus kakipanjang	Wostl, E., Riyanto, A., Hamidy, A., Kurniawan, N., Smith, E. N. and Harvey, M. B. (2017). A taxonomic revision of the *Philautus* (Anura: Rhacophoridae) of Sumatra with the description of four new species. *Herpetological Monographs* 31: 98–141.
Philautus kerange	Wostl, E., Riyanto, A., Hamidy, A., Kurniawan, N., Smith, E. N. and Harvey, M. B. (2017). A taxonomic revision of the *Philautus* (Anura: Rhacophoridae) of Sumatra with the description of four new species. *Herpetological Monographs* 31: 98–141.
Philautus leitensis	Brown, W. C., and Alcala, A. C. (1994). Philippine frogs of the family Rhacophoridae. *Proceedings of the California Academy of Sciences*, *4th Series* 48: 185–220.
Philautus longicrus	Dehling, J. M. (2010). A new bush frog (Anura: Rhacophoridae: *Philautus*) from Gunung Mulu National Park, East Malaysia (Borneo). *Salamandra* 46: 63–72.
Philautus nephophilus	Wostl, E., Riyanto, A., Hamidy, A., Kurniawan, N., Smith, E. N. and Harvey, M. B. (2017). A taxonomic revision of the *Philautus* (Anura: Rhacophoridae) of Sumatra with the description of four new species. *Herpetological Monographs* 31: 98–141.
Philautus nianeae	Stuart, B. L., Phimmachak, S., Seateun, S. and Sheridan, J. A. (2013). A new *Philautus* (Anura: Rhacophoridae) from northern Laos allied to *P. abditus* Inger, Orlov & Darevsky, 1999. *Zootaxa* 3745: 73–83.
Philautus pallidipes	Bossuyt, F., and Dubois, A. (2001). A review of the frog genus *Philautus* Gistel, 1848 (Amphibia, Anura, Ranidae, Rhacophorinae). *Zeylanica* 6: 1–112.
Philautus poecilius	Brown, W. C., and Alcala, A. C. (1994). Philippine frogs of the family Rhacophoridae. *Proceedings of the California Academy of Sciences*, *4th Series* 48: 185–220.
Philautus saueri	Malkmus, R., and Riede, K. (1996). Die Baumfrösche der Gattung *Philautus* vom Mount Kinabalu—Teil I: Überblick und die *aurifasciatus*-Gruppe mit Beschreibung einer neuen Art (*Philautus saueri* n. sp.). *Sauria* 18: 27–37.
Philautus schmackeri	Brown, W. C., and Alcala, A. C. (1994). Philippine frogs of the family Rhacophoridae. *Proceedings of the California Academy of Sciences*, *4th Series* 48: 185–220.
Philautus surudus	Wostl, E., Riyanto, A., Hamidy, A., Kurniawan, N., Smith, E. N. and Harvey, M. B. (2017). A taxonomic revision of the *Philautus* (Anura: Rhacophoridae) of Sumatra with the description of four new species. *Herpetological Monographs* 31: 98–141.
Philautus surrufus	Brown, W. C., and Alcala, A. C. (1994). Philippine frogs of the family Rhacophoridae. *Proceedings of the California Academy of Sciences*, *4th Series* 48: 185–220.
Philautus tytthus	Smith, M. A. (1940). The amphibians and reptiles obtained by Mr. Ronald

	Kaulback in Upper Burma. *Records of the Indian Museum* 42: 465–486.
Polypedates assamensis	Mathew, R., and Sen, N. (2009). Studies on little known amphibians of Northeast India. *Records of the Zoological Survey of India*. *Occasional Papers* 293: 1–64, 23
Polypedates hecticus	Brown, R. M., Linkem, C. W., Siler, C. D., Sukumaran, J., Esselstyn, J. A., Diesmos, A. C., Iskandar, D. T., Bickford, D. P., Evans, B. J., McGuire, J. A., Grismer, L. L., Supriatna, J. and Andayani, N. (2010). Phylogeography and historical demography of *Polypedates leucomystax* in the islands of Indonesia and the Philippines: Evidence for recent human-mediated range expansion? *Molecular Phylogenetics and Evolution* 57: 598–619.
Polypedates himalayaensis	Mathew, R., and Sen, N. (2009). Studies on little known amphibians of Northeast India. *Records of the Zoological Survey of India*. *Occasional Papers* 293: 1–64, 23
Polypedates insularus	Das, I. (1995). A new species of tree frog (genus *Polypedates*) from Great Nicobar, India (Anura: Rhacophoridae). *Hamadryad* 20: 13–20.
Polypedates iskandari	Riyanto, A., Mumpuni, A. and McGuire, J. A. (2011). Morphometry of striped tree frogs, *Polypedates leucomystax* (Gravenhorst, 1829) from Indonesia with description of a new species. *Russian Journal of Herpetology* 18: 29–35.
Polypedates pseudotilophus	Matsui, M., Hamidy, A. and Kuraishi, N. (2014). A new species of *Polypedates* from Sumatra, Indonesia (Amphibia: Anura). *Species Diversity* 19: 1–7.
Polypedates ranwellai	Wickramasinghe, L. J. M., Munindradasa, D. A. I. and Fernando, P. (2012). A new species of *Polypedates* Tschudi (Amphibia, Anura, Rhacophoridae) from Sri Lanka. *Zootaxa* 3498: 63–80.
Polypedates subansiriensis	Mathew, R., and Sen, N. (2009). Studies on little known amphibians of Northeast India. *Records of the Zoological Survey of India*. *Occasional Papers* 293: 1–64, 23
Polypedates taeniatus	Dubois, A. (1987) "1986". Miscellanea taxinomica batrachologica (I). *Alytes* Paris 5: 7–95.
Polypedates zed	Anders, C. C. (2002). Class Amphibia (Amphibians). Schleich, H. H., and W. Kästle (Ed)., Amphibians and Reptiles of Nepal: Biology, Systematics, Field Guide: 133–340. Ruggell, A.R.G. Gantner Verlag K.G.
Pseudophilautus hypomelas	Manamendra-Arachchi, K., and Pethiyagoda, R. (2005). The Sri Lankan shrub-frogs of the genus *Philautus* Gistel, 1848 (Ranidae: Rhacophorinae), with description of 27 new species. Contributions to Biodiversity Exploration and Research in Sri Lanka. *Raffles Bulletin of Zoology*, Supplement 12: 163–303.
Pseudophilautus nemus	Manamendra-Arachchi, K., and Pethiyagoda, R. (2005). The Sri Lankan shrub-frogs of the genus *Philautus* Gistel, 1848 (Ranidae: Rhacophorinae), with description of 27 new species. Contributions to Biodiversity Exploration and Research in Sri Lanka. *Raffles Bulletin of Zoology*, Supplement 12: 163–303.
Raorchestes sahai	Sarkar, A. K., and Ray, S. (2006). Amphibia. Alfred, J. R. B. (Ed)., Fauna of

	Arunachal Pradesh. Part 1.: Calcutta, *Zoological Survey of India*.
Raorchestes anandalii	Bossuyt, F., and Dubois, A. (2001). A review of the frog genus *Philautus* Gistel, 1848 (Amphibia, Anura, Ranidae, Rhacophorinae). *Zeylanica* 6: 1–112.
Rhacophorus barisani	Harvey, M. B., Pemberton, A. J. and Smith, E. N. (2002). New and poorly known parachuting frogs (Rhacophoridae: *Rhacophorus*) from Sumatra and Java. *Herpetological Monographs* 16: 46–92.
Rhacophorus laoshan	Mo, Y. M., Jiang, J. P., Xie, F. and Ohler, A. (2008). A new species of *Rhacophorus* (Anura: Ranidae) from China. *Asiatic Herpetological Research* 11: 85–92.
Rhacophorus larissae	Ostroshabov, A. A., Orlov, N. L. and Nguyen, T. T. (2013). Taxonomy of frogs of genus *Rhacophorus* of "*hoanglienensis–orlovi*" complex. *Russian Journal of Herpetology* 20: 301–324.
Rhacophorus marmoridorsum	Orlov, N. L. (2008). Description of a new species of *Rhacophorus* genus (Amphibia: Anura: Rhacophoridae) from Kon Cha Rang area (Gia Lai Province, Vietnam). *Russian Journal of Herpetology* 15: 133–140.
Theloderma nagalandense	Poyarkov, N. A., Orlov, Jr., N. L., Moiseeva, A. V., Pawangkhanant, P., Ruangsuwan, T., Vassilieva, A. B., Galoyan, E. A., Nguyen, T. T. and Gogoleva, S. I. (2015). Sorting out Moss Frogs: mtDNA data on taxonomic diversity and phylogenetic relationships of the Indochinese species of the genus *Theloderma* (Anura, Rhacophoridae). *Russian Journal of Herpetology* 22: 241–280.
Zhangixalus achantharrhena	Harvey, M. B., Pemberton, A. J. and Smith, E. N. (2002). New and poorly known parachuting frogs (Rhacophoridae: *Rhacophorus*) from Sumatra and Java. *Herpetological Monographs* 16: 46–92.
Zhangixalus arvalis	Fei, L., Hu, S. Q., Ye, C. Y and Huang, Y. Z. (2009). *Fauna Sinica*. Amphibia. Volume 2. Anura. Beijing: Chinese Academy of Science. Science Press.
Zhangixalus aurantiventris	Fei, L., Hu, S. Q., Ye, C. Y and Huang, Y. Z. (2009). *Fauna Sinica*. Amphibia. Volume 2. Anura. Beijing: Chinese Academy of Science. Science Press.
Zhangixalus prasinatus	Fei, L., Hu, S. Q., Ye, C. Y and Huang, Y. Z. (2009). *Fauna Sinica*. Amphibia. Volume 2. Anura. Beijing: Chinese Academy of Science. Science Press.
Zhangixalus taipeianus	Dubois, A. (1987) "1986". Miscellanea taxinomica batrachologica (I). *Alytes* Paris 5: 7–95.
Zhangixalus yinggelingensis	Fei, L., Hu, S. Q., Ye, C. Y and Huang, Y. Z. (2009). *Fauna Sinica*. Amphibia. Volume 2. Anura. Beijing: Chinese Academy of Science. Science Press.

Table 9. Statistical results from BioGeoBEARS. Comparison of the fit of different models of geographic-range evolution and model-specific estimates for the different parameters. The models are Dispersal-Extinction-Cladogenesis (DEC); ML version of Dispersal-Vicariance Analysis (DIVALIKE); and Bayesian biogeographical inference model (BAYAREALIKE), each with and without founder-event speciation (+J). Abbreviations: LnL, log-likelihood; d, rate of dispersal; e, rate of extinction; j, likelihood of founder-event speciation at cladogenesis; AIC, Akaike's information criterion. The AIC model weight is used to compare all the models together and select the best one. The preferred model is indicated by bold red font.

Model	LnL	numparams	d	e	j	AICc	AICc_wt
DEC	-268.1	2	0.0092	1.00E-12	0	540.3	1.30E-19
**DEC+J**	**-223.7**	**3**	**0.0025**	**1.00E-12**	**0.067**	**453.5**	**0.92**
DIVALIKE	-269.2	2	0.013	1.00E-12	0	542.5	4.40E-20
DIVALIKE+J	-226.2	3	0.0032	1.00E-12	0.066	458.5	0.077
BAYAREALIKE	-417.8	2	0.01	0.01	0	839.6	1.40E-84
BAYAREALIKE+J	-231.8	3	0.002	1.00E-07	0.071	469.6	0.0003
