# Supplementary material for: The role of climate and islands in species diversification and reproductive-mode evolution of Old World tree frogs
Source: Commun Biol. 2022 Apr 11;5:347. doi: 10.1038/s42003-022-03292-1 (PMC9001633; doi:10.1038/s42003-022-03292-1)
Supplement: Supplementary file 2 — Description of Additional Supplementary Files [file 42003_2022_3292_MOESM2_ESM.pdf]

Description of Additional Supplementary Files

File name: Supplementary Data 1
Description: Mean values for each bioclimatic variable for each species

File name: Supplementary Data 2
Description: A set of 1000 post burn-in topologies of the rhacophorid phylogeny

File name: Supplementary Data 3
Description: The maximum clade credibility tree for Rhacophoridae

File name: Supplementary Data 4
Description: Source data
