# Supplementary material for: The role of climate and islands in species diversification and reproductive-mode evolution of Old World tree frogs
Source: Commun Biol. 2022 Apr 11;5:347. doi: 10.1038/s42003-022-03292-1 (PMC9001633; doi:10.1038/s42003-022-03292-1)
Supplement: Supplementary file 7 — Reporting Summary [file 42003_2022_3292_MOESM7_ESM.pdf]

nature portfolio

Corresponding author(s): Madhava Meegaskumbura
COMMSBIO-21-2530A

Last updated by author(s): Feb 25, 2022

Reporting Summary

Nature Portfolio wishes to improve the reproducibility of the work that we publish. This form provides structure for consistency and transparency in reporting. For further information on Nature Portfolio policies, see our Editorial Policies and the Editorial Policy Checklist.

Statistics

For all statistical analyses, confirm that the following items are present in the figure legend, table legend, main text, or Methods section.

n/a	Confirmed	
☐	☒	The exact sample size (*n*) for each experimental group/condition, given as a discrete number and unit of measurement
☒	☐	A statement on whether measurements were taken from distinct samples or whether the same sample was measured repeatedly
☐	☒	The statistical test(s) used AND whether they are one- or two-sided<br>*Only common tests should be described solely by name; describe more complex techniques in the Methods section.*
☐	☒	A description of all covariates tested
☐	☒	A description of any assumptions or corrections, such as tests of normality and adjustment for multiple comparisons
☐	☒	A full description of the statistical parameters including central tendency (e.g. means) or other basic estimates (e.g. regression coefficient) AND variation (e.g. standard deviation) or associated estimates of uncertainty (e.g. confidence intervals)
☐	☒	For null hypothesis testing, the test statistic (e.g. *F*, *t*, *r*) with confidence intervals, effect sizes, degrees of freedom and *P* value noted<br>*Give P values as exact values whenever suitable.*
☐	☒	For Bayesian analysis, information on the choice of priors and Markov chain Monte Carlo settings
☒	☐	For hierarchical and complex designs, identification of the appropriate level for tests and full reporting of outcomes
☒	☐	Estimates of effect sizes (e.g. Cohen's *d*, Pearson's *r*), indicating how they were calculated

Our web collection on statistics for biologists contains articles on many of the points above.

Software and code

Policy information about availability of computer code

Data collection

Taxonomic data on Rhacophoridae obtained from;
AmphibiaWeb. Information on amphibian biology and conservation. University of California, Berkeley, California: http://www.amphibiaweb.org/ (2020) and
Frost, D. R. Amphibian Species of the World. An Online Reference. Version 6.1 https://amphibiansoftheworld.amnh.org/index.php (2020)

Anchor Hybrid Enrichment Data set (AHE) of Rhacophoridae, available at https://datadryad.org/stash/dataset/doi:10.5061/dryad.2jm63xsk6 was downloaded;
Chen, J.-M. et al. An integrative phylogenomic approach illuminates the evolutionary history of Old World tree frogs (Anura: Rhacophoridae). Mol. Phylog. Evol. 145, 106724.

Nucleotide data deposited in GenBank (last accessed January 2021) which is available at https://www.ncbi.nlm.nih.gov/genbank/ were downloaded (Accession numbers provided in Supplementary Table 7)

The distribution ranges of all rhacophorid species obtained as spatial data polygons from IUCN (2020) http://www.iucnredlist.org/technical-documents/spatial-data;
IUCN (International Union for Conservation of Nature) 2019. Rhacophoridae. The IUCN Red List of Threatened Species. Version 2020-3. http://www.iucnredlist.org.

Species occurrence records obtained from the GBIF database (https://www.gbif.org) using code provided at RGBIF 1.4.0;
Chamberlain, S. et al. rgbif: Interface to the Global Biodiversity Information Facility API. R package version 0.9.8 (2020). Available at https://CRAN.Rproject.org/package=rgbif

Information on 19 bioclimatic variables for each species occurrence point were obtained from WORLDCLIM 2.0;

Fick, S. E. & Hijmans, R. J. WorldClim 2: new 1km spatial resolution climate surfaces for global land areas. Int. J. Climatol. 37, 4302–4315 (2017)

Data analysis

RAxML 8.2.12;
Stamatakis, A. RAxML version 8: a tool for phylogenetic analysis and post-analysis of large phylogenies. Bioinformatics 30, 1312–1313 (2014).

ASTRAL III v.5.7.3
Zhang, C., Rabiee, M., Sayyari, E. & Mirarab, S. ASTRAL-III: polynomial time species tree reconstruction from partially resolved gene trees. BMC Bioinf. 19, 153 (2018).

MUSCLE
Edgar, R. C. MUSCLE: a multiple sequence alignment method with reduced time and space complexity. BMC Bioinf. 5, 113 (2004).

MEGA v.6
Tamura, K., Stecher, G., Peterson, D., Filipski, A. & Kumar, S. MEGA6: Molecular Evolutionary Genetics Analysis Version 6.0. Mol. Biol. Evol. 30, 2725–2729 (2013).

PASTIS
Thomas, G. H. et al. PASTIS: an R package to facilitate phylogenetic assembly with soft taxonomic inferences. Meth. Ecol. Evol. 4, 1011–1017 (2013).

MrBayes 3.2.5
Ronquist, F. et al. MrBayes 3.2: Efficient Bayesian phylogenetic inference and model choice across a large model space. Syst. Biol. 61, 539–542 (2012).

APE 5.4
Paradis, E., Claude, J. & Strimmer, K. APE: Analyses of phylogenetics and evolution in R language. Bioinformatics 20, 289–290 (2004).

CAPER 0.2
Orme, D. et al. caper: Comparative Analyses of Phylogenetics and Evolution in R version 0.5. (2018). Available from: http://CRAN.R-project.org/package=caper

PartitionFinder2
Lanfear, R., Frandsen, P. B., Wright, A. M., Senfeld, T. & Calcott, B. PartitionFinder 2: New methods for selecting partitioned models of evolution for molecular and morphological phylogenetic analyses. Mol. Biol. Evol. msw260 (2016) doi:10.1093/molbev/msw260

TRACER v.1.6
Drummond, A. J., Suchard, M. A., Xie, D. & Rambaut, A. Bayesian phylogenetics with BEAUti and the BEAST 1.7. Mol. Biol. Evol. 29, 1969–1973 (2012).

TREEANNOTATOR v.1.10.4
Rambaut, A. & Drummond, A. J. TreeAnnotator v1.10.4. Available as part of the BEAST package ahttp://beast.bio.ed.ac.ukt . (2013).

ES-sim implemented using the code available at https://github.com/mgharvey/ES-sim
Harvey, M. G. & Rabosky, D. L. Continuous traits and speciation rates: Alternatives to state_dependent diversification models. Meth. Ecol. Evol. 9, 984–993 (2018).

PHYTOOLS 0.7-47
Revell, L. J. phytools: an R package for phylogenetic comparative biology (and other things). Meth. Ecol. Evol. 3, 217–223 (2012).

letsR V 3.2
Vilela, B. & Villalobos, F. letsR: a new R package for data handling and analysis in macroecology. Meth. Ecol. Evol. 6, 1229–1234 (2015).

RPANDA
Morlon, H. et al. RPANDA: an R package for macroevolutionary analyses on phylogenetic trees. Meth. Ecol. Evol. 7, 589–597 (2016).

RASTER 3.0-7
Hijmans, R. J. et al. raster: Geographic Data Analysis and Modeling (R package). (2020). Available at https://CRAN.R-project.org/package=raster

VEGAN 2.5-6
Oksanen, J. et al. vegan: Community Ecology Package. (2019). Available at https://github.com/vegandevs/vegan

GEIGER 2.0.6.2
Harmon, L. J., Weir, J. T., Brock, C. D., Glor, R. E. & Challenger, W. GEIGER: investigating evolutionary radiations. Bioinformatics 24, 129–131 (2008).

BioGeoBEARS v.0.2.1
Matzke, N. J., BioGeoBEARS: BioGeography with Bayesian (and Likelihood) evolutionary analysis in R scripts. R package, version 0.2.1 (2013).

Nichevolve v.0.1.19
Owens, H. L. et al. Acknowledging uncertainty in evolutionary reconstructions of ecological niches. Ecol. Evol. 10, 6967–6977 (2020)

climateStability v.0.1.3
Owens, H. L. & Guralnick, R. climateStability: An R package to estimate climate stability from time-slice climatologies. Biodiv Inf. 14, 8–13 (2014).

CANAPE
Mishler, B. D. et al. Phylogenetic measures of biodiversity and neo- and paleo-endemism in Australian Acacia. Nat. Commun. 5, 4473 (2014).

For manuscripts utilizing custom algorithms or software that are central to the research but not yet described in published literature, software must be made available to editors and reviewers. We strongly encourage code deposition in a community repository (e.g. GitHub). See the Nature Portfolio guidelines for submitting code & software for further information.

Data

Policy information about availability of data

All manuscripts must include a data availability statement. This statement should provide the following information, where applicable:

- Accession codes, unique identifiers, or web links for publicly available datasets
- A description of any restrictions on data availability
- For clinical datasets or third party data, please ensure that the statement adheres to our policy

The maximum clade credibility tree, a set of 1000 post burn-in topologies and species means of bioclimatic variables are provided in Supplementary Data 1. For Genbank accession numbers see Supplementary Table 7. Additional data that support the findings of this study are publicly available online at www.amphibiaweb.org, https://amphibiansoftheworld.amnh.org/index.php, https://datadryad.org/stash/dataset/doi:10.5061/dryad.2jm63xsk6, https://www.ncbi.nlm.nih.gov/genbank/, http://www.iucnredlist.org/technical-documents/spatial-data, https://www.gbif.org and https://www.worldclim.org.

Field-specific reporting

Please select the one below that is the best fit for your research. If you are not sure, read the appropriate sections before making your selection.

☐ Life sciences ☐ Behavioural & social sciences ☒ Ecological, evolutionary & environmental sciences

For a reference copy of the document with all sections, see nature.com/documents/nr-reporting-summary-flat.pdf

Life sciences study design

All studies must disclose on these points even when the disclosure is negative.

Sample size	*Describe how sample size was determined, detailing any statistical methods used to predetermine sample size OR if no sample-size calculation was performed, describe how sample sizes were chosen and provide a rationale for why these sample sizes are sufficient.*
Data exclusions	*Describe any data exclusions. If no data were excluded from the analyses, state so OR if data were excluded, describe the exclusions and the rationale behind them, indicating whether exclusion criteria were pre-established.*
Replication	*Describe the measures taken to verify the reproducibility of the experimental findings. If all attempts at replication were successful, confirm this OR if there are any findings that were not replicated or cannot be reproduced, note this and describe why.*
Randomization	*Describe how samples/organisms/participants were allocated into experimental groups. If allocation was not random, describe how covariates were controlled OR if this is not relevant to your study, explain why.*
Blinding	*Describe whether the investigators were blinded to group allocation during data collection and/or analysis. If blinding was not possible, describe why OR explain why blinding was not relevant to your study.*

Behavioural & social sciences study design

All studies must disclose on these points even when the disclosure is negative.

Study description	*Briefly describe the study type including whether data are quantitative, qualitative, or mixed-methods (e.g. qualitative cross-sectional, quantitative experimental, mixed-methods case study).*
Research sample	*State the research sample (e.g. Harvard university undergraduates, villagers in rural India) and provide relevant demographic information (e.g. age, sex) and indicate whether the sample is representative. Provide a rationale for the study sample chosen. For studies involving existing datasets, please describe the dataset and source.*
Sampling strategy	*Describe the sampling procedure (e.g. random, snowball, stratified, convenience). Describe the statistical methods that were used to predetermine sample size OR if no sample-size calculation was performed, describe how sample sizes were chosen and provide a rationale for why these sample sizes are sufficient. For qualitative data, please indicate whether data saturation was considered, and what criteria were used to decide that no further sampling was needed.*
Data collection	*Provide details about the data collection procedure, including the instruments or devices used to record the data (e.g. pen and paper, computer, eye tracker, video or audio equipment) whether anyone was present besides the participant(s) and the researcher, and whether the researcher was blind to experimental condition and/or the study hypothesis during data collection.*
Timing	*Indicate the start and stop dates of data collection. If there is a gap between collection periods, state the dates for each sample cohort.*

Data exclusions	*If no data were excluded from the analyses, state so OR if data were excluded, provide the exact number of exclusions and the rationale behind them, indicating whether exclusion criteria were pre-established.*
Non-participation	*State how many participants dropped out/declined participation and the reason(s) given OR provide response rate OR state that no participants dropped out/declined participation.*
Randomization	*If participants were not allocated into experimental groups, state so OR describe how participants were allocated to groups, and if allocation was not random, describe how covariates were controlled.*

Ecological, evolutionary & environmental sciences study design

All studies must disclose on these points even when the disclosure is negative.

Study description	Analyses of the evolutionary impacts of climate are often based on predictions about distributions that use ecological niche models in the context of climatic conservatism. In such cases, lineages are thought to maintain their climatic niche through evolutionary timescales. This can lead to false conclusions because lineages can also adapt (evolve) to exploit certain climatic conditions. Hence, here we test both how lineages track optimal climatic conditions and also how they adapt to new conditions. We use phylogenetic imputations to construct comprehensive phylogenies of a widely distributed tree frog family (Rhacophoridae; comprising 6% of global amphibians), trait grams,bin-based ancestral state reconstructions and disparity through time plots to identify species exhibiting climatic niche evolution and higher rates of evolution. We perform clade-level analyses to show the extent to which derived clades have shifted from the climatic niches of early-emerging clades and explain the role of Niche Evolution vs. Niche conservatism for each clade in Rhacophoridae. We also identify regions with relatively high diversification rates such as islands and archipelagos and discuss how increased ecological opportunity is achieved by these frogs. Further, we show that reproductive mode evolution is correlated with specific climatic events such that we conclude, starting from a centre of origin, rhacophorids reached distant refugia either by adapting to new climatic conditions ('niche evolution'-dominant), especially following the origin of key innovations such as terrestrial reproduction (in the Late Eocene) or by dispersal during periods of favourable climate ('niche conservatism'-dominant).
Research sample	We use the large diversification of old world tree frogs (family Rhacophoridae), which represent 6% of the global amphibians as our research sample.<br><br>Some diversifications are spread across small geographic areas, where differences in climate are modest. To test hypotheses on climatic adaptation through evolutionary time, however, old, widespread diversifications are preferred. Therefore, we concentrate on a large group of climatically sensitive (ectothermic) tree frogs (rhacophorids) constituting 6% of the global amphibian diversity, that are spread across a large geographic area (Asia and Africa: from temperate, subtropical to tropical, including continental islands) to test hypotheses on how climate influences diversification and dispersal.
Sampling strategy	Analyses of large diversifications are often handicapped by incomplete taxon sampling. Usually extensive taxon sampling is important in phylogenetic systematics as it increases accuracy and support of evolutionary relationships. Therefore we employed a recently developed method, taxonomic imputation, while also using a combination of available genomic and Sanger sequence data to construct the most comprehensive phylogeny of Rhacophoridae up to date. Thereby, we construct a 415-species tree for rhacophorids representing all 22 genera. In addition when obtaining bioclimatic data we used the highest resolution available (30arcsec) to increase the accuracy of our data extraction process.
Data collection	All data were obtained from publicly available data repositories which are listed in the references section.
Timing and spatial scale	Our phylogenetic tree represent the entire diversification of Rhacophoridae which evolved around 68-53mya during early Eocene
Data exclusions	Some nucleotide data were removed which resulted in chimeric sequences. In addition few of the occurrence data obtained from GBIF were excluded due to duplication of points and which provided NA values after extraction of data from Raster maps. We assumed that these NA values may have resulted due to data points which were located just outside coastal boundaries which are often caused by errors in GPS readings. The RGBIF provided 4473 geographical coordinates for Rhacophoridae, however the final distribution dataset comprised 3846 geographical coordinates of all species which were used in subsequent analyses.
Reproducibility	All attempts to repeat the analyses were successful
Randomization	Species were allocated into groups based on previously published literature.<br>Biogeographical categorizations were based on Li et al., (2013) and Chen et al., (2020);<br>Li, J.-T. et al. Diversification of rhacophorid frogs provides evidence for accelerated faunal exchange between India and Eurasia during the Oligocene. Proc. Natl. Acad. Sci. U.S.A. 110, 3441–3446 (2013).<br>Chen, J.-M. et al. An integrative phylogenomic approach illuminates the evolutionary history of Old World tree frogs (Anura: Rhacophoridae). Mol. Phylog. Evol. 145, 106724 (2020).<br><br>The reproductive modes categorizations were based on Meegaskumbura et al., (2015).<br>Meegaskumbura, M. et al. Patterns of reproductive-mode evolution in Old World tree frogs (Anura, Rhacophoridae). Zool. Scr. 44, 509–522 (2015).
Blinding	Not relevant to this study as we do not deal with experimental animals. This study deals with already published data available in public repositories.

Did the study involve field work? ☐ Yes ☒ No

Field work, collection and transport

Field conditions	*Describe the study conditions for field work, providing relevant parameters (e.g. temperature, rainfall).*
Location	*State the location of the sampling or experiment, providing relevant parameters (e.g. latitude and longitude, elevation, water depth).*
Access & import/export	*Describe the efforts you have made to access habitats and to collect and import/export your samples in a responsible manner and in compliance with local, national and international laws, noting any permits that were obtained (give the name of the issuing authority, the date of issue, and any identifying information).*
Disturbance	*Describe any disturbance caused by the study and how it was minimized.*

Reporting for specific materials, systems and methods

We require information from authors about some types of materials, experimental systems and methods used in many studies. Here, indicate whether each material, system or method listed is relevant to your study. If you are not sure if a list item applies to your research, read the appropriate section before selecting a response.

Materials & experimental systems

n/a	Involved in the study	
☒	☐	Antibodies
☒	☐	Eukaryotic cell lines
☒	☐	Palaeontology and archaeology
☒	☐	Animals and other organisms
☒	☐	Human research participants
☒	☐	Clinical data
☒	☐	Dual use research of concern

Methods

n/a	Involved in the study	
☒	☐	ChIP-seq
☒	☐	Flow cytometry
☒	☐	MRI-based neuroimaging

Antibodies

Antibodies used	*Describe all antibodies used in the study; as applicable, provide supplier name, catalog number, clone name, and lot number.*
Validation	*Describe the validation of each primary antibody for the species and application, noting any validation statements on the manufacturer's website, relevant citations, antibody profiles in online databases, or data provided in the manuscript.*

Eukaryotic cell lines

Policy information about cell lines

Cell line source(s)	*State the source of each cell line used.*
Authentication	*Describe the authentication procedures for each cell line used OR declare that none of the cell lines used were authenticated.*
Mycoplasma contamination	*Confirm that all cell lines tested negative for mycoplasma contamination OR describe the results of the testing for mycoplasma contamination OR declare that the cell lines were not tested for mycoplasma contamination.*
Commonly misidentified lines (See ICLAC register)	*Name any commonly misidentified cell lines used in the study and provide a rationale for their use.*

Palaeontology and Archaeology

Specimen provenance	*Provide provenance information for specimens and describe permits that were obtained for the work (including the name of the issuing authority, the date of issue, and any identifying information). Permits should encompass collection and, where applicable, export.*
Specimen deposition	*Indicate where the specimens have been deposited to permit free access by other researchers.*
Dating methods	*If new dates are provided, describe how they were obtained (e.g. collection, storage, sample pretreatment and measurement), where they were obtained (i.e. lab name), the calibration program and the protocol for quality assurance OR state that no new dates are provided.*

☐ Tick this box to confirm that the raw and calibrated dates are available in the paper or in Supplementary Information.

Ethics oversight	*Identify the organization(s) that approved or provided guidance on the study protocol, OR state that no ethical approval or guidance was required and explain why not.*

Note that full information on the approval of the study protocol must also be provided in the manuscript.

Animals and other organisms

Policy information about studies involving animals; ARRIVE guidelines recommended for reporting animal research

Laboratory animals	*For laboratory animals, report species, strain, sex and age OR state that the study did not involve laboratory animals.*
Wild animals	*Provide details on animals observed in or captured in the field; report species, sex and age where possible. Describe how animals were caught and transported and what happened to captive animals after the study (if killed, explain why and describe method; if released, say where and when) OR state that the study did not involve wild animals.*
Field-collected samples	*For laboratory work with field-collected samples, describe all relevant parameters such as housing, maintenance, temperature, photoperiod and end-of-experiment protocol OR state that the study did not involve samples collected from the field.*
Ethics oversight	*Identify the organization(s) that approved or provided guidance on the study protocol, OR state that no ethical approval or guidance was required and explain why not.*

Note that full information on the approval of the study protocol must also be provided in the manuscript.

Human research participants

Policy information about studies involving human research participants

Population characteristics	*Describe the covariate-relevant population characteristics of the human research participants (e.g. age, gender, genotypic information, past and current diagnosis and treatment categories). If you filled out the behavioural & social sciences study design questions and have nothing to add here, write "See above."*
Recruitment	*Describe how participants were recruited. Outline any potential self-selection bias or other biases that may be present and how these are likely to impact results.*
Ethics oversight	*Identify the organization(s) that approved the study protocol.*

Note that full information on the approval of the study protocol must also be provided in the manuscript.

Clinical data

Policy information about clinical studies

All manuscripts should comply with the ICMJE guidelines for publication of clinical research and a completed CONSORT checklist must be included with all submissions.

Clinical trial registration	*Provide the trial registration number from ClinicalTrials.gov or an equivalent agency.*
Study protocol	*Note where the full trial protocol can be accessed OR if not available, explain why.*
Data collection	*Describe the settings and locales of data collection, noting the time periods of recruitment and data collection.*
Outcomes	*Describe how you pre-defined primary and secondary outcome measures and how you assessed these measures.*

Dual use research of concern

Policy information about dual use research of concern

Hazards

Could the accidental, deliberate or reckless misuse of agents or technologies generated in the work, or the application of information presented in the manuscript, pose a threat to:

No	Yes	
☐	☐	Public health
☐	☐	National security
☐	☐	Crops and/or livestock
☐	☐	Ecosystems
☐	☐	Any other significant area

Experiments of concern

Does the work involve any of these experiments of concern:

No	Yes	
☐	☐	Demonstrate how to render a vaccine ineffective
☐	☐	Confer resistance to therapeutically useful antibiotics or antiviral agents
☐	☐	Enhance the virulence of a pathogen or render a nonpathogen virulent
☐	☐	Increase transmissibility of a pathogen
☐	☐	Alter the host range of a pathogen
☐	☐	Enable evasion of diagnostic/detection modalities
☐	☐	Enable the weaponization of a biological agent or toxin
☐	☐	Any other potentially harmful combination of experiments and agents

ChIP-seq

Data deposition

☐ Confirm that both raw and final processed data have been deposited in a public database such as GEO.

☐ Confirm that you have deposited or provided access to graph files (e.g. BED files) for the called peaks.

Data access links<br>*May remain private before publication.*	*For "Initial submission" or "Revised version" documents, provide reviewer access links. For your "Final submission" document, provide a link to the deposited data.*
Files in database submission	*Provide a list of all files available in the database submission.*
Genome browser session<br>(e.g. UCSC)	*Provide a link to an anonymized genome browser session for "Initial submission" and "Revised version" documents only, to enable peer review. Write "no longer applicable" for "Final submission" documents.*

Methodology

Replicates	*Describe the experimental replicates, specifying number, type and replicate agreement.*
Sequencing depth	*Describe the sequencing depth for each experiment, providing the total number of reads, uniquely mapped reads, length of reads and whether they were paired- or single-end.*
Antibodies	*Describe the antibodies used for the ChIP-seq experiments; as applicable, provide supplier name, catalog number, clone name, and lot number.*
Peak calling parameters	*Specify the command line program and parameters used for read mapping and peak calling, including the ChIP, control and index files used.*
Data quality	*Describe the methods used to ensure data quality in full detail, including how many peaks are at FDR 5% and above 5-fold enrichment.*
Software	*Describe the software used to collect and analyze the ChIP-seq data. For custom code that has been deposited into a community repository, provide accession details.*

Flow Cytometry

Plots

Confirm that:

☐ The axis labels state the marker and fluorochrome used (e.g. CD4-FITC).

☐ The axis scales are clearly visible. Include numbers along axes only for bottom left plot of group (a 'group' is an analysis of identical markers).

☐ All plots are contour plots with outliers or pseudocolor plots.

☐ A numerical value for number of cells or percentage (with statistics) is provided.

Methodology

Sample preparation	*Describe the sample preparation, detailing the biological source of the cells and any tissue processing steps used.*
Instrument	*Identify the instrument used for data collection, specifying make and model number.*

Software	*Describe the software used to collect and analyze the flow cytometry data. For custom code that has been deposited into a community repository, provide accession details.*
Cell population abundance	*Describe the abundance of the relevant cell populations within post-sort fractions, providing details on the purity of the samples and how it was determined.*
Gating strategy	*Describe the gating strategy used for all relevant experiments, specifying the preliminary FSC/SSC gates of the starting cell population, indicating where boundaries between "positive" and "negative" staining cell populations are defined.*

☐ Tick this box to confirm that a figure exemplifying the gating strategy is provided in the Supplementary Information.

Magnetic resonance imaging

Experimental design

Design type	*Indicate task or resting state; event-related or block design.*
Design specifications	*Specify the number of blocks, trials or experimental units per session and/or subject, and specify the length of each trial or block (if trials are blocked) and interval between trials.*
Behavioral performance measures	*State number and/or type of variables recorded (e.g. correct button press, response time) and what statistics were used to establish that the subjects were performing the task as expected (e.g. mean, range, and/or standard deviation across subjects).*

Acquisition

Imaging type(s)	*Specify: functional, structural, diffusion, perfusion.*
Field strength	*Specify in Tesla*
Sequence & imaging parameters	*Specify the pulse sequence type (gradient echo, spin echo, etc.), imaging type (EPI, spiral, etc.), field of view, matrix size, slice thickness, orientation and TE/TR/flip angle.*
Area of acquisition	*State whether a whole brain scan was used OR define the area of acquisition, describing how the region was determined.*

Diffusion MRI ☐ Used ☐ Not used

Preprocessing

Preprocessing software	*Provide detail on software version and revision number and on specific parameters (model/functions, brain extraction, segmentation, smoothing kernel size, etc.).*
Normalization	*If data were normalized/standardized, describe the approach(es): specify linear or non-linear and define image types used for transformation OR indicate that data were not normalized and explain rationale for lack of normalization.*
Normalization template	*Describe the template used for normalization/transformation, specifying subject space or group standardized space (e.g. original Talairach, MNI305, ICBM152) OR indicate that the data were not normalized.*
Noise and artifact removal	*Describe your procedure(s) for artifact and structured noise removal, specifying motion parameters, tissue signals and physiological signals (heart rate, respiration).*
Volume censoring	*Define your software and/or method and criteria for volume censoring, and state the extent of such censoring.*

Statistical modeling & inference

Model type and settings	*Specify type (mass univariate, multivariate, RSA, predictive, etc.) and describe essential details of the model at the first and second levels (e.g. fixed, random or mixed effects; drift or auto-correlation).*
Effect(s) tested	*Define precise effect in terms of the task or stimulus conditions instead of psychological concepts and indicate whether ANOVA or factorial designs were used.*

Specify type of analysis: ☐ Whole brain ☐ ROI-based ☐ Both

Statistic type for inference (See Eklund et al. 2016)	*Specify voxel-wise or cluster-wise and report all relevant parameters for cluster-wise methods.*
Correction	*Describe the type of correction and how it is obtained for multiple comparisons (e.g. FWE, FDR, permutation or Monte Carlo).*

Models & analysis

n/a	Involved in the study
☒	☐ Functional and/or effective connectivity
☒	☐ Graph analysis
☒	☐ Multivariate modeling or predictive analysis
